# Acute and subacute toxicity evaluation of ZhenzhuXiaoji decoction in preclinical models: implications for safe clinical use

**DOI:** 10.3389/fphar.2025.1554732

**Published:** 2025-04-15

**Authors:** Songzhe Li, Shuchang Bao, Lingyun Cai, Baolong Li, Yue Sun, Yang Sun

**Affiliations:** ^1^ Department of Biology, College of Basic Medicine, Heilongjiang University of Chinese Medicine, Harbin, China; ^2^ Nanjing Seventeen Retirement Center for Retired Cadres, Nanjing, China

**Keywords:** ZhenzhuXiaoji decoction, traditional Chinese medicine, mechanism, toxicology, pharmacology, hepatoenal toxichety

## Abstract

**Background:**

ZhenzhuXiaoji Decoction (ZZXJD) is a traditional Chinese medicine formulation composed of five herbs: *Ligustrum lucidum*, *Curcuma zedoaria*, *Prunella vulgaris*, *Hedyotis diffusa*, and *Glycyrrhiza uralensis*, developed for the treatment of hepatocellular carcinoma (HCC). Although early studies have demonstrated the therapeutic potential of ZZXJD, its safety profile, particularly regarding potential toxicity, remains underexplored. This study aims to evaluate both the pharmacological effects and toxicity of ZZXJD in preclinical models to determine its clinical applicability.

**Study design and Methods:**

This study employed *in vitro* and *in vivo* experiments to assess the pharmacological effects and safety of ZZXJD. HHL-5 and HEK-293 cell lines were treated with ZZXJD at varying concentrations (125, 250, 500, and 1,000 μg/mL) for 24, 48, and 72 h to evaluate its effects on cell viability, apoptosis, and necrosis. Acute and subacute toxicity studies were conducted in male and female mice, including assessments of behavioral changes, body weight, organ weight, and liver/kidney functions. Additionally, routine blood tests were performed to identify potential immunostimulatory effects.

**Results:**

*In vitro* experiments demonstrated that ZZXJD inhibited the proliferation of HHL-5 and HEK-293 cells in a dose-dependent manner and induced apoptosis and necrosis. In subacute toxicity studies, mice in the low and mid-dose groups exhibited no significant behavioral changes, whereas the high-dose group showed transient alterations in liver and kidney function markers, particularly in female mice. These changes were reversible following treatment cessation. Blood tests indicated increased lymphocyte and monocyte counts in treated male mice; however, these increases were not statistically significant. Organ weight and histopathological analyses revealed no significant signs of toxicity at therapeutic doses.

**Conclusion:**

Treatment with ZZXJD at standard therapeutic dosage did not produce acute or subacute toxic effects on liver or kidney functions *in vivo*, suggesting its safety for continued use in cancer treatment. However, reversible abnormalities in liver and kidney function markers were observed at higher doses. Thus, regular monitoring of liver and kidney functions is recommended during clinical use, especially when higher doses are employed.

## 1 Introduction

Traditional Chinese Medicine (TCM) has a long history of application in China, playing a crucial role in treating various diseases (Kai et al., 2021; Wu et al., 2021). With the advancement of modern pharmaceutical systems, several clinically effective TCM medicines have been developed into patented formulations, which are now widely used in clinical practice (Jia et al., 2015; Wang et al., 2019). Based on specific symptomatic presentations and principles underlying herbal formulations, many novel combinations have also been developed (Song et al., 2019). Nevertheless, even clinically validated traditional prescriptions may occasionally produce adverse reactions during clinical use (Xu et al., 2016). Historically, most newly developed medicines have stalled prior to reaching clinical application, primarily due to uncontrollable toxic side effects, which remain the leading cause of their failure (Brodniewicz and Grynkiewicz, 2010).

Zhenzhu Xiaoji Decoction (ZZXJD) is a clinical formulation prescribed to inhibit hepatocellular carcinoma (HCC). ZZXJD comprises five herbal components: *Ligustrum lucidum*, *Curcuma zedoaria*, *Prunella vulgaris*, *Hedyotis diffusa*, and *Glycyrrhiza uralensis*. Previous studies have demonstrated that ZZXJD induces autophagy and apoptosis in hepatocellular carcinoma cells via the AKT/mTOR and JAK2/STAT3 signaling pathways, resulting in programmed cell death and inhibition of HCC progression (Sun et al., 2022). Additionally, a gene chip analysis combined with bioinformatics has indicated that the FOXO signaling pathway may also represent a potential mechanism underlying ZZXJD’s anti-cancer effects (Li et al., 2022); however, conclusive evidence is currently lacking. In summary, ZZXJD’s anti-HCC activity has been preliminarily confirmed, although its potential toxic effects during treatment have not yet been clarified due to an absence of toxicological studies.

This study conducted acute and 28-day subacute toxicity experiments in rodents, referencing therapeutic dosages from previous clinical and mouse studies (Sun et al., 2022). The aim was to collect toxicity data on ZZXJD to establish a foundational safety profile for future animal and clinical research. Additionally, the active components and their respective concentrations in ZZXJD were identified via LC-MS analysis, providing essential theoretical support for the further development of the formulation.

## 2 Materials and methods

### 2.1 Experimental medicine

ZZXJD was provided by the First Affiliated Hospital of Heilongjiang University of Chinese Medicine. The herbal components were tested and authenticated to ensure quality and consistency (Sun et al., 2022). Authentication was performed by pharmacognosy experts from Heilongjiang University of Chinese Medicine, according to standardized protocols outlined in the *Pharmacopoeia of the People’s Republic of China* (2020 edition). All herbal samples were securely stored in the Department of Biology, School of Basic Medical Sciences, Heilongjiang University of Chinese Medicine.

### 2.2 Preparation of experimental drugs

The water decoction method was used to prepare a single dose of ZZXJD (125 g). Initially, water was added to cover the medicinal herbs. After the first decoction, additional water was added three times to the herbal residue, and the resulting supernatant was collected each time. The combined supernatants were thoroughly stirred, mixed, and filtered to remove herbal residues, and then concentrated by heating at 65°C for 90 min. Finally, the concentrated extract was placed on a tray and freeze-dried overnight at −80 °C using a freeze dryer (SCIENTZ-12N/D, Ningbo, China) to eliminate moisture. The total weight of the herbal medicine before decoction was 125 g, and the resulting powder weighed approximately 15.2 g after freeze-drying. The freeze-dried powder was mixed into the appropriate culture medium and filtered using a 0.22 μm membrane filter. A clear stock solution was finally prepared at a concentration of 1 mg/mL.

### 2.3 Chemical composition analysis of ZZXJD

The extract of ZZXJD was measured by KAITAI-BIO (Zhejiang, China). Accurately weigh an appropriate amount of sample into a 2 mL centrifuge tube, add 600 µL MeOH (Containing 2-Amino-3-(2-chloro-phenyl)-propionic acid (4 ppm), vortex for 30 s; Add steel balls, placed in a tissue grinder for 60 s at 55 Hz; Room temperature ultrasound for 15 min; Centrifuge for 10 min at 12,000 rpm and 4°C, filter the supernatant by 0.22 μm membrane and transfer into the detection bottle for LC-MS detection (Vasilev et al., 2016).

#### 2.3.1 Liquid chromatography conditions

The LC analysis was performed on a Vanquish UHPLC System (Thermo Fisher Scientific, United States). Chromatography was carried out with an ACQUITY UPLC ^®^ HSS T3 (2.1 × 100 mm, 1.8 µm) (Waters, Milford, MA, United States). The column maintained at 40 °C. The flow rate and injection volume were set at 0.3 mL/min and 2 μL, respectively. For LC-ESI (+)-MS analysis, the mobile phases consisted of (B2) 0.1% formic acid in acetonitrile (v/v) and (A2) 0.1% formic acid in water (v/v). Separation was conducted under the following gradient: 0∼1 min, 8% B2; 1∼8 min, 8%∼98% B2; 8∼10 min, 98% B2; 10∼10.1 min, 98%∼8% B2; 10.1∼12 min, 8% B2. For LC-ESI (−)-MS analysis, the analytes was carried out with (B3) acetonitrile and (A3) ammonium formate (5 mM). Separation was conducted under the following gradient: 0∼1 min,8% B3; 1∼8 min, 8%∼98% B3; 8∼10 min,98% B3; 10∼10.1 min,98%∼8% B3; 10.1∼12 min, 8%B3 (Development of a robust and repeatable UPLC-MS method for the long-term metabolomic study of human serum) (Zelena et al., 2009).

#### 2.3.2 Mass spectrum conditions

Mass spectrometric detection of metabolites was performed on Q Exactive (Thermo Fisher Scientific, United States) with ESI ion source. Simultaneous MS1 and MS/MS (Full MS-ddMS2 mode, data-dependent MS/MS) acquisition was used. The parameters were as follows: sheath gas pressure, 40 arb; aux gas flow, 10 arb; spray voltage, 3.50 kV and −2.50 kV for ESI(+) and ESI(−), respectively; capillary temperature, 325 °C; MS1 range, m/z 100–1,000; MS1 resolving power, 70,000 FWHM; number of data dependant scans per cycle, 10; MS/MS resolving power, 17,500 FWHM; normalized collision energy, 30 eV; dynamic exclusion time, automatic (Global metabolic profiling of animal and human tissues via UPLC-MS) (Ej et al., 2013).

### 2.4 *In vitro* experiments

#### 2.4.1 Cell culture

HEK-293 cells (Otwo Biotech, Shenzhen, China) were cultured in Dulbecco’s Modified Eagle Medium (DMEM) supplemented with 10% fetal bovine serum and 1% penicillin/streptomycin. HHL-5 cells (Otwo Biotech, Shenzhen, China) were cultured in RPMI-1640 medium supplemented with 10% fetal bovine serum and 1% penicillin/streptomycin. Both cell types were seeded and passed into 25 cm^2^ culture flasks. When the cells reached 80%–90% confluence, they were digested and centrifuged. After centrifugation, the supernatant was discarded, and fresh medium was added. After passaging, cells were incubated at 37 °C with 5% CO_2_ to continue culturing.

#### 2.4.2 Cytotoxicity test

Hepatic and renal toxicity are among the potential risks associated with Traditional Chinese Medicine. To assess the potential toxicity of ZZXJD and better understand its risk profile, ZZXJD was administered to HHL-5 and HEK-293 cells, both of which are frequently employed for *in vitro* toxicity evaluations (Zhou et al., 2021; Fatemeh et al., 2023). A Cell Counting Kit-8 (CCK8; Meilunbio, Dalian, China) assay was used to determine the inhibitory effect of ZZXJD. Each cell line was seeded into 96-well plates and incubated overnight to ensure adherence. The following day, the old medium was replaced with fresh medium containing ZZXJD at concentrations of 0, 125, 250, 500, and 1,000 μg/mL, and cells were incubated for 24, 48, or 72 h. After each incubation period, 10 μL of CCK8 solution was added to each well, and plates were returned to the incubator for an additional 2 h. Subsequently, the optical density (OD) at 450 nm was measured using a microplate reader (Infinite M200 PRO; TECAN, Mannedorf, Switzerland) employing the single-well multiple detection method. Each experiment was repeated three times, and the cell inhibition rate was calculated using the following formula: Inhibition rate (%) = [(OD _control–OD _experimental)/(OD _control–OD _blank)] × 100%.

#### 2.4.3 Cell morphology staining

Cells were stained using a double staining method with Hoechst 33,342 (Meilunbio, Dalian, China) and propidium iodide (PI; Sigma, Shanghai, China). Stock solutions of 1 mg/mL Hoechst 33,342 and 2 mg/mL PI were used. Cell slides were placed into the wells of a 24-well plate, and cell suspensions of each cell line were prepared. The cell suspensions were then added onto the slides, followed by incubation for 30 min. After initial incubation, 1 mL of the corresponding medium for each cell line was added, and slides were incubated overnight. The following day, the old culture medium was replaced with fresh medium containing ZZXJD at concentrations ranging from 0 to 1,000 μg/mL. After 24 h of incubation, the supernatant was discarded, and the slides were washed three times with phosphate-buffered saline (PBS). Finally, 1 mL of PBS containing 5 μL Hoechst 33,342 and 2.5 μL PI was added to each well, and the slides were incubated for 20 min. Cells were then observed and photographed within 1 h using a fluorescence microscope (EVOS FL Auto, Life Technologies, Carlsbad, SD, United States).

### 2.5 Acute toxicity test

A total of 28 eight-week-old, specific-pathogen-free (SPF)-grade institute of Cancer Research (ICR) mice (21–29 g; 14 males and 14 females) were purchased from Liaoning Changsheng (SCXK 2020-0001). All mice were housed in an SPF animal laboratory at 23°C ± 2 °C with 50% ± 10% humidity under a 12 h light/dark cycle, provided with free access to food and water, and acclimated for 7 days. The study protocol was reviewed and approved by the Chinese Medicine Institutional Animal Care and Use Committee of Heilongjiang University (No. 2021060301). The mice were randomly divided into four groups according to sex and treatment: female control, female treated, male control, and male treated. Male and female mice in each group were housed separately.

This experiment was conducted according to the Organization for Economic Co-operation and Development (OECD) Guideline 420 (OECD 420). Prior to ZZXJD administration, all ICR mice were weighed and fasted for 12 h while maintaining free access to water. Based on preliminary experimental results, mice in the treatment group received a single oral gavage dose of ZZXJD at 10,000 mg/kg (freeze-dried powder per body weight). ZZXJD was dissolved in double-distilled water and administered at a volume of 20 mL/kg; the control group received an equal volume of double-distilled water. Mice in the treatment group were dosed sequentially, and their general condition and potential toxicity signs were closely monitored for 1 h post-administration. Irrespective of the survival of the first mouse, the second mouse received an identical dose. If the second mouse survived, the procedure continued similarly with the third mouse and subsequent mice. However, if the first two mice died, the experiment was terminated immediately. Food was reintroduced 4 h after dosing. Mice were continuously monitored for behavioral changes, signs of toxicity, and mortality over a period of 14 days, during which they were allowed free access to food and water. On day 14, all surviving mice were weighed, and the median lethal dose (LD_50_) was calculated.

### 2.6 Subacute toxicity experiment

Sixty mice were randomly divided into 12 groups (5 mice per each), balanced for sex: control, low-dose, medium-dose, high-dose, control satellite, and high-dose satellite groups. Each group contained an equal number of male and half female mice. Male and female mice in each group were housed separately.

The OECD Guideline 407 was employed to guide this experiment. Mice were weighed, and body weights were recorded prior to treatment. The ZZXJD treatment was administered orally at low, medium, and high doses (2.08 g/kg, 4.16 g/kg, and 8.32 g/kg, respectively). ZZXJD was dissolved in double-distilled water and administered by oral gavage at a volume of 10 mL/kg; the control group received an equal volume of double-distilled water. Treatment continued daily for 28 consecutive days, after which the mice were euthanized and dissected. To assess delayed toxicity or the reversibility of toxic effects, additional control satellite and high-dose satellite groups were established. These satellite groups received the same doses and treatment duration as the control and high-dose groups, respectively, followed by an additional 14-day recovery period without treatment. At the end of the recovery period, mice were euthanized and dissected. All mice were weighed prior to euthanasia, and the LD_50_ was calculated.

### 2.7 Blood tests

At the end of the experiment, mice were weighed and sedated by intraperitoneal injection of ketamine hydrochloride (80 mg/kg). Following sedation, mice were anesthetized by intraperitoneal injection of sodium pentobarbital (40 mg/kg). Blood samples were collected by retro-orbital bleeding into EDTA-containing anticoagulant tubes. After blood collection, the mice were euthanized via intraperitoneal injection of sodium pentobarbital (120 mg/kg). Blood samples were analyzed using an automated hematology analyzer (XS-500i; Sysmex, Japan). The measured parameters included white blood cell (WBC), lymphocyte (LYMPH), monocyte (MONO), basophils (BASO), LYMPH%, MONO%, BASO%, red blood cell (RBC), hemoglobin (HGB), hematocrit (HCT), mean corpusular volume (MCV), mean corpusular hemoglobin (MCH), mean corpusular hemoglobin concerntration (MCHC), red blood cell distribution width coefficient of variation (RDW-CV), red blood cell distribution width-standard deviation (RDW-SD), platelet (PLT) and bone alkaline phosphatase (BALP).

### 2.8 Measurement of liver and kidney biochemical parameters

A portion of the collected blood was transferred into sterile centrifuge tubes and left undisturbed for 4 h. Serum was then separated by centrifugation at 3,000 rpm for 15 min at low temperature. The serum samples were analyzed using an automatic biochemical immunoassay analyzer (Cobas 8000 702–1; Roche, Switzerland). The measured biochemical parameters included total bilirubin (TBil), direct bilirubin (DBil), total protein (TPro), albumin (ALB), aspartate aminotransferase (AST), alanine aminotransferase (ALT), total bile acid (TBA), urea nitrogen (BUN), inosine (CRE), and uric acid (UA).

### 2.9 Relative organ weight measurement

During necropsy, the liver, kidneys, spleen, testes, epididymides, heart, seminal vesicles, ovaries, uterus, adrenal glands, bladder, brain, and thymus were collected and individually weighed. After weighing, organs were fixed by immersion in 4% paraformaldehyde solution. Relative organ weights were calculated using the following formula: relative organ weight (%) = [organ weight (g)/mouse body weight (g)] × 100.

### 2.10 Paraffin embedding of tissue

Testicular tissue was fixed in Davidson’s fixative (Leagene, Beijing, China), while the remaining tissues were fixed in 4% paraformaldehyde for 48 h. Fixed tissue were rinsed under running water to remove excess fixative, dehydrated through graded alcohol solutions, cleared in xylene, and infiltrated with paraffin wax in an incubator. After infiltration, the tissues were embedded into paraffin blocks, trimmed to appropriate sizes, and stored until sectioning.

### 2.11 HE staining

We used a tissue microtome (RM2135, Leica, Germany) to section the paraffin-embedded tissue blocks into slices approximately 6 μm thick. Tissue sections were treated twice with xylene (FuYu, Tianjin, China) and anhydrous alcohol (FuYu, Tianjin, China) for 5 min each, followed by gradient alcohol solutions for 5 min. Sections were then immersed in distilled water for 5 min, stained with hematoxylin (HE; alcohol-soluble; HuiShi, Shanghai, China) for 10 min, and rinsed with tap water. After differentiation in 70% alcohol containing 1% hydrochloric acid, sections were again rinsed with tap water for 5 min. Next, the tissue sections underwent dehydration through graded alcohol and were immersed in 90% alcoholic eosin solution for 8 min. Finally, sections were dehydrated twice in anhydrous alcohol and cleared twice in xylene. Slides were mounted using neutral gum and photographed at ×200 magnification with a photomicrography imaging system (Moticam 3,000; San Antonio, TX, United States).

### 2.12 Statistical analysis

All statistical analyses were performed using SPSS 26.0 software. Data conforming to a normal distribution were analyzed using one-way ANOVA, while data not conforming to a normal distribution were analyzed using nonparametric tests. All data are expressed as mean ± standard deviation (mean ± SD). Differences were considered statistically significant at *P* < 0.05 and highly significant at *P* < 0.01.

## 3 Results

### 3.1 LC-MS component determination

**FIGURE 1:**
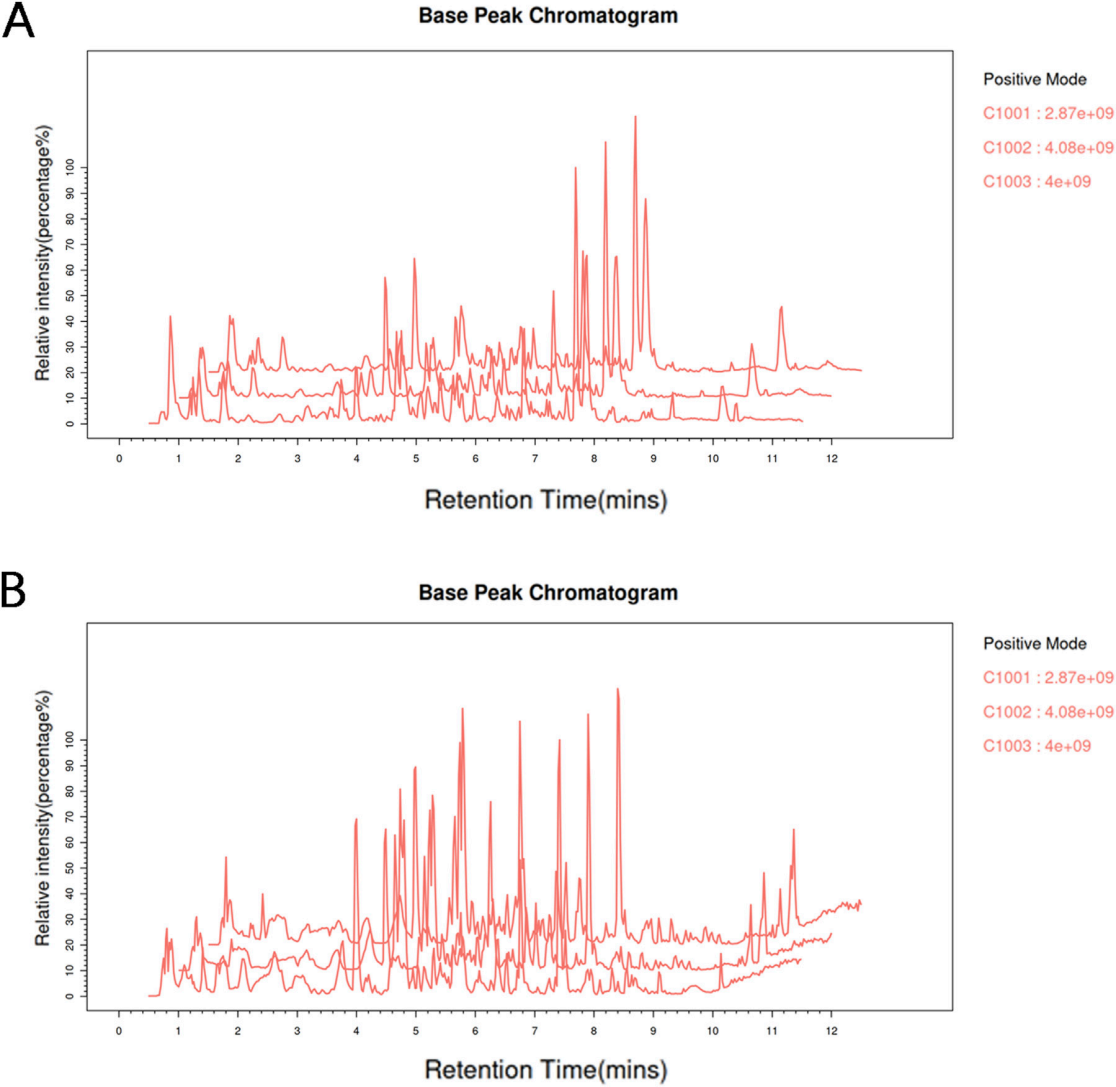
Total base peak chromatograms. Note: **(A)**: Total base peak chromatogram of the sample in the positive ion mode. **(B)**: Total base peak chromatogram of the sample in negative ion mode.

The total ion chromatograms (TIC) of ZZXJD samples analyzed in positive and negative ion modes are presented in Figure 1. The identified components and their concentrations in ZZXJD are summarized in Supplementary Table S1.

### 3.2 *In vitro* experiments

#### 3.2.1 Short-term inhibitory effects of ZZXJD on liver and kidney cells

**FIGURE 2:**
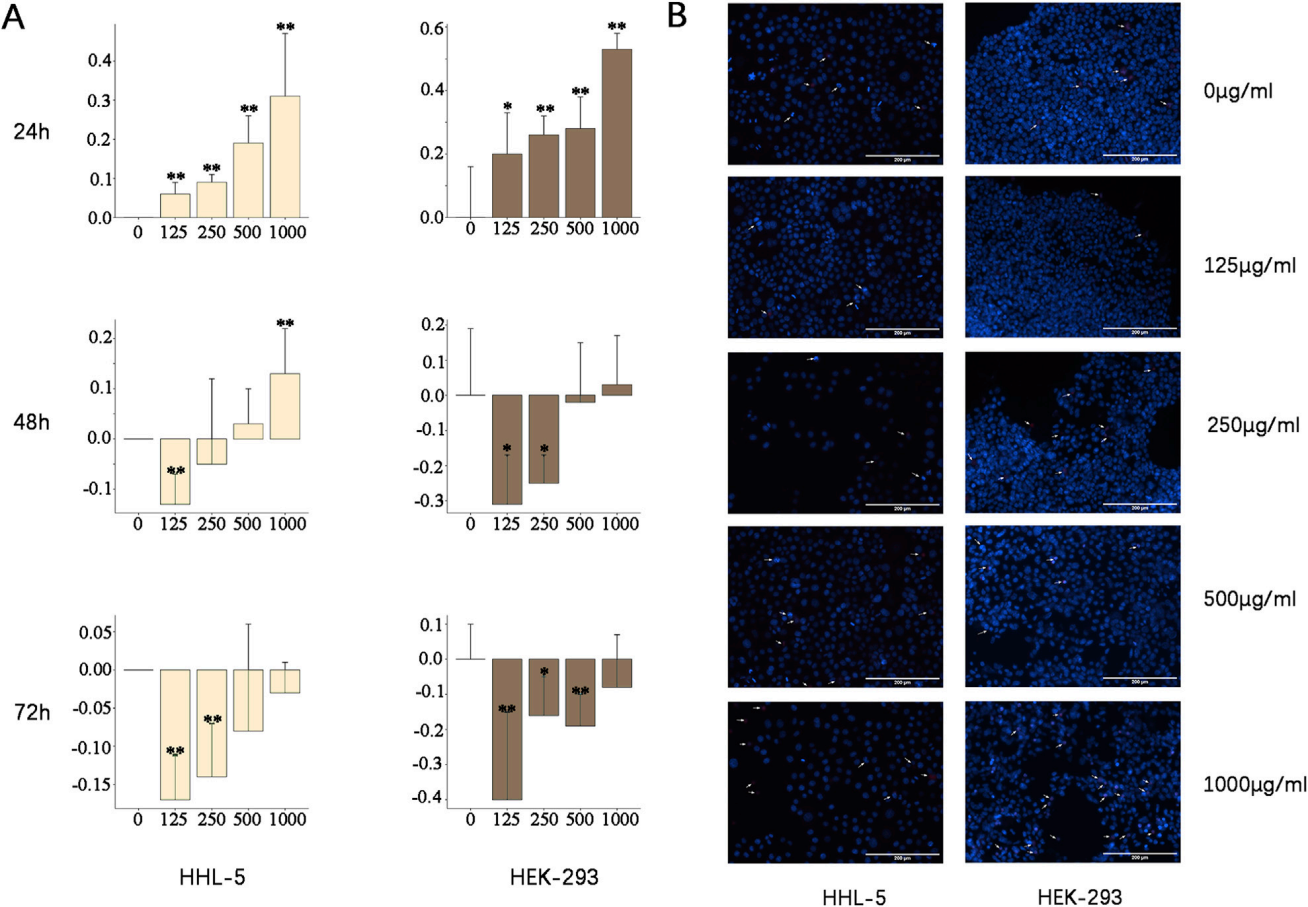
*In vitro* experiments Note: Compared to 0 μg/mL, *: *P* < 0.05. * *: *P* < 0.01. **(A)** Inhibitory rates (%) of different concentrations of ZZXJD on HHL-5 and HEK-293 cells at 24, 48, and 72 h **(B)** Scale bar = 200 µm. According to the reagent descriptions, apoptotic cells exhibit strong blue fluorescence, whereas advanced apoptotic and necrotic cells exhibit strong red and strong blue fluorescence.

ZZXJD exhibited a certain degree of inhibitory effect on HHL-5 and HEK-293 cells at 24 h. However, a gradual proliferative effect was observed after 48 h (Figure 2A).

#### 3.2.2 ZZXJD promotes apoptosis and necrosis

Given that ZZXJD exhibited varying degrees of inhibitory effects on both cell lines at 24 h, Hoechst 33342/PI fluorescence double staining was performed to observe morphological changes in these cells following 24 h of medicine treatment. The staining results revealed that both cell lines underwent apoptosis and necrosis to varying extents, with a general increase correlating with higher drug concentrations (Figure 2B). At concentrations of 125, 250, and 500 μg/mL, apoptosis was notably elevated in HHL-5 cells, whereas HEK-293 cells exhibited more pronounced apoptosis and necrosis. At the concentration of 1,000 μg/mL, a significant increase in apoptotic and necrotic cells was observed in both cell types.

### 3.3 Acute toxicity test

The acute toxicity experiment revealed that some mice showed decreased activity within 1 h following oral gavage administration, but no other evident signs of toxicity were observed. Mice resumed feeding normally within 4 h after treatment, and no abnormal symptoms were noted thereafter. Throughout the study period, no significant behavioral or pathological differences were observed between the control and treatment groups. All mice survived until the completion of the experiment, suggesting an LD_50_ greater than 10 g/kg. According to toxicity classification criteria, ZZXJD exhibited no acute toxicity. With regard to body weight changes, mice displayed varying degrees of weight gain, and no significant differences were observed between male and female mice. Furthermore, food and water intake correlated positively with body weight increases. Necropsy examinations revealed no significant pathological alterations attributable to ZZXJD treatment, and relative organ weights did not differ significantly between the control and ZZXJD-treated groups.

### 3.4 Subacute toxicity test

#### 3.4.1 Behavioral, body weight, and food intake changes

During the treatment period, mice in the low-dose and mid-dose groups exhibited no abnormal behavioral changes following treatment. However, female mice in the high-dose and high-dose satellite groups displayed reduced activity after dosing, whereas no abnormal behaviors were observed in the corresponding male mice. All mice survived for the duration of the experiment. Dull fur was commonly observed in female mice from the high-dose and high-dose satellite groups, and some mice in the mid-dose group also exhibited this condition. After discontinuation of treatment, the dull fur condition in female mice of the high-dose satellite group markedly improved. Some male mice in the high-dose and high-dose satellite groups similarly exhibited dull fur, although this condition notably improved in male mice of the high-dose satellite group following cessation of treatment. Regarding body weight, all mouse groups showed overall weight gain, with similar weight-gain trends observed between male and female mice. Additionally, food and water consumption trends were consistent between sexes, with no significant abnormalities noted.

#### 3.4.2 Blood tests results

Blood test results are presented in Table 1. Compared with the control group, abnormalities were observed in male but not female mice, as evidenced by varying degrees of elevation in lymphocyte and monocyte counts following ZZXJD treatment. Statistically significant differences were found in the high-dose or mid-dose groups. In the satellite groups, lymphocyte counts were elevated in treated male mice compared to controls; however, no significant differences were observed for other parameters in either male or female mice.

**TABLE 1:** Blood test results (n = 5).

Parameter	Control (F/M)	Low (F/M)	Medium (F/M)	High (F/M)	Satellite	
					Control (F/M)	High (F/M)
WBC	3.12 ± 1.57/2.51 ± 0.73	4.76 ± 0.81/3.43 ± 1.07	4.52 ± 1.28/3.76 ± 1.44	4.91 ± 2.04/4.26 ± 1.79	2.96 ± 1.06/1.78 ± 1.18	2.89 ± 0.68/3.90 ± 0.83
LYMPH	2.62 ± 1.35/2.09 ± 0.61	4.17 ± 0.88/2.79 ± 0.63	3.81 ± 1.12/3.18 ± 1.33	4.40 ± 1.82/3.73 ± 1.69^*^	2.59 ± 1.05/1.45 ± 1.11	2.52 ± 0.61/3.46 ± 0.69
MONO	0.03 ± 0.02/0.02 ± 0.02	0.02 ± 0.02/0.03 ± 0.01	0.17 ± 0.28/0.14 ± 0.18^*^	0.05 ± 0.02/0.04 ± 0.04	0.03 ± 0.03/0.06 ± 0.09	0.02 ± 0.01/0.03 ± 0.02
BASO	0.17 ± 0.11/0.15 ± 0.12	0.30 ± 0.07/0.15 ± 0.06	0.27 ± 0.11/0.15 ± 0.05	0.24 ± 0.10/0.17 ± 0.04	0.19 ± 0.03/0.09 ± 0.02	0.17 ± 0.04/0.27 ± 0.12
LYMPH%	84.48 ± 6.93/83.26 ± 3.43	87.14 ± 4.09/86.04 ± 3.84	84.64 ± 6.11/84.34 ± 6.77	89.60 ± 2.93/86.66 ± 4.04	85.96 ± 5.82/79.00 ± 11.67	86.92 ± 3.11/88.90 ± 2.21
MONO%	0.78 ± 0.63/0.94 ± 0.56	0.52 ± 0.47/0.92 ± 0.38	3.68 ± 5.95/4.02 ± 5.82	1.08 ± 0.54/1.08 ± 1.02	0.86 ± 0.72/4.30 ± 6.87	0.66 ± 0.50/0.70 ± 0.44
BASO%	5.80 ± 3.03/6.94 ± 6.54	6.56 ± 2.55/5.26 ± 3.02	6.08 ± 1.86/3.94 ± 0.44	5.20 ± 2.83/4.76 ± 2.38	7.68 ± 3.96/6.46 ± 2.91	5.76 ± 0.50/6.74 ± 1.92
RBC	11.43 ± 0.61/11.35 ± 1.29	11.55 ± 1.70/11.56 ± 0.85	11.12 ± 0.94/11.02 ± 1.58	11.80 ± 0.38/11.30 ± 1.09	10.78 ± 1.37/11.32 ± 1.14	11.80 ± 0.69/11.32 ± 1.32
HGB	158.60 ± 8.56/153.60 ± 18.43	157.60 ± 25.21/158.00 ± 14.46	155.40 ± 8.02/149.60 ± 18.65	163.00 ± 6.89/154.80 ± 16.04	152.00 ± 22.42/154.20 ± 12.30	163.20 ± 5.12/150.40 ± 15.24
HCT	56.18 ± 0.44/54.88 ± 5.24	55.88 ± 7.91/55.42 ± 3.99	57.66 ± 3.58/54.82 ± 5.85	58.10 ± 1.38/57.00 ± 4.53	54.72 ± 7.93/56.60 ± 2.03	58.08 ± 1.65/54.92 ± 5.34
MCV	49.10 ± 2.84/48.44 ± 1.77	48.48 ± 2.29/47.98 ± 1.95	51.96 ± 1.56/50.00 ± 2.36	50.08 ± 2.76/50.50 ± 1.29	50.66 ± 2.26/50.32 ± 4.21	49.26 ± 1.80/48.62 ± 2.29
MCH	13.88 ± 0.31/13.54 ± 0.53	13.64 ± 0.59/13.66 ± 0.48	14.02 ± 0.83/13.60 ± 0.35	13.42 ± 0.59/13.70 ± 0.19	14.06 ± 0.51/13.64 ± 0.56	13.86 ± 0.54/13.32 ± 0.41
MCHC	282.40 ± 10.69/279.20 ± 7.82	281.40 ± 7.13/284.80 ± 8.98	270.00 ± 14.93/272.40 ± 6.80	268.60 ± 21.55/271.20 ± 9.07	277.80 ± 7.36/272.00 ± 13.04	281.00 ± 6.04/273.60 ± 4.83
RDW-CV	28.38 ± 1.97/27.96 ± 1.70	28.40 ± 2.84/28.74 ± 0.96	26.10 ± 0.77/26.78 ± 2.34	27.48 ± 5.09/26.82 ± 1.41	26.42 ± 1.34/27.60 ± 2.82	28.08 ± 1.36/27.70 ± 2.12
RDW-SD	40.40 ± 2.85/38.30 ± 1.57	38.76 ± 3.45/39.38 ± 1.43	36.58 ± 2.05/37.78 ± 1.79	40.10 ± 5.02/37.70 ± 0.91	37.32 ± 0.83/39.46 ± 1.81	38.40 ± 0.65/37.64 ± 1.72
PLT	426.40 ± 291.01/186.00 ± 195.97	549.40 ± 204.75/634.00 ± 234.36^**^	335.80 ± 255.82/299.00 ± 187.63	177.20 ± 81.30/202.00 ± 54.82	490.00 ± 174.54/262.80 ± 176.76	422.40 ± 366.04/338.60 ± 218.69
BALP	0.01 ± 0.01/0.01 ± 0.02	0.02 ± 0.02/0.04 ± 0.05	0.05 ± 0.04/0.10 ± 0.09	0.07 ± 0.07/0.09 ± 0.09	0.06 ± 0.05/0.11 ± 0.10	0.10 ± 0.09/0.02 ± 0.02

Note: Compared with sex-matched controls within each group, *: *P* < 0.05. * *: *P* < 0.01. F: female. M: male.

#### 3.4.3 Test results of liver and kidney biochemical indicators

The results of liver and kidney function tests are presented in Table 2. Compared with the control group, ZZXJD treatment resulted in abnormalities in certain hepatic and renal parameters. Female mice exhibited more pronounced liver and kidney abnormalities than male mice, while male mice primarily showed changes in kidney parameters. Liver and kidney abnormalities in both male and female mice increased with escalating doses of ZZXJD. In the satellite groups, no significant differences in hepatic or renal parameters were found between treated and control mice of either sex.

**TABLE 2:** Hepatic and renal biochemical parameter results (n = 5).

Parameter	Control (F/M)	Low (F/M)	Medium (F/M)	High (F/M)	Satellite	
					Control (F/M)	High (F/M)
TBil	1.96 ± 0.69/6.87 ± 5.70	2.61 ± 0.49/4.42 ± 1.37	3.47 ± 1.89^*^/5.37 ± 2.24	2.06 ± 0.80/5.60 ± 1.40	1.93 ± 0.34/4.01 ± 1.06	1.76 ± 0.19/3.70 ± 0.54
DBil	1.10 ± 0.35/3.85 ± 3.23	1.29 ± 0.26/2.34 ± 0.78	2.11 ± 1.30^*^/3.08 ± 1.09	1.23 ± 0.33/3.47 ± 0.79	1.06 ± 0.16/2.31 ± 0.74	1.14 ± 0.18/1.92 ± 0.25
TPro	61.06 ± 3.19/60.11 ± 3.58	61.16 ± 4.42/60.36 ± 2.21	59.36 ± 2.18/57.36 ± 3.18	55.48 ± 2.63^*^/56.78 ± 3.15	61.89 ± 2.52/58.50 ± 4.02	60.34 ± 1.67/57.97 ± 1.30
ALB	45.94 ± 2.12/42.04 ± 2.38	44.69 ± 2.79/41.05 ± 2.46	44.85 ± 3.70/41.41 ± 1.51	42.38 ± 1.84^**^/39.92 ± 3.21	45.35 ± 0.86/40.63 ± 2.44	45.44 ± 1.88/40.66 ± 1.74
AST	217.80 ± 28.13/209.40 ± 54.66	191.80 ± 37.49^*^/190.20 ± 41.56	161.20 ± 44.30^**^/193.20 ± 30.47	141.40 ± 24.54/175.60 ± 30.15	188.80 ± 38.60/216.20 ± 24.66	195.80 ± 37.31/213.80 ± 55.51
ALT	54.20 ± 10.28/62.60 ± 27.79	47.80 ± 23.97/44.20 ± 13.63	45.40 ± 3.85/46.60 ± 14.21	34.60 ± 5.13^*^/49.40 ± 10.11	51.60 ± 5.59/65.00 ± 15.75	46.80 ± 7.79/54.20 ± 6.42
TBA	12.71 ± 8.06/41.97 ± 16.78	23.38 ± 11.06/19.97 ± 18.43	20.98 ± 9.09/13.29 ± 5.38	56.74 ± 103.57/13.99 ± 7.48	9.76 ± 3.69/12.35 ± 6.72	19.81 ± 9.03/6.48 ± 1.76
BUN	9.14 ± 1.97/8.54 ± 1.05	6.56 ± 0.48^**^/8.32 ± 1.69	7.64 ± 1.06/6.68 ± 1.25^*^	6.78 ± 1.20^*^/7.26 ± 1.32	8.30 ± 2.13/8.78 ± 1.53	7.66 ± 1.08/8.12 ± 1.69
CRE	7.20 ± 0.45/6.20 ± 0.84	6.20 ± 0.45^*^/5.80 ± 0.84	6.20 ± 0.45^*^/7.00 ± 1.41	5.80 ± 1.30^*^/7.20 ± 1.10	9.40 ± 0.89/7.20 ± 1.64	9.00 ± 0.00/7.40 ± 1.14
UA	225.67 ± 94.37/178.25 ± 16.40	149.88 ± 19.39^*^/172.56 ± 26.05	137.37 ± 29.47^*^/136.30 ± 6.87^**^	144.60 ± 27.98^*^/134.55 ± 7.27^**^	193.40 ± 71.72/168.80 ± 29.49	160.43 ± 83.33/163.20 ± 21.41

Note: Compared with sex-matched controls within each group, *: *P* < 0.05. * *: *P* < 0.01. F: female. M: male.

#### 3.4.4 Relative organ weight measurement results

Results for relative organ weights (Table 3) indicated a significant increase in the relative weights of seminal vesicles and testes in male mice treated with ZZXJD compared with the control group (*P* < 0.05). No other significant differences in relative organ weights were detected among the remaining male and female groups.

**TABLE 3:** Relative organ weights of mice in the subacute toxicity study (n = 5).

Organ	Control (F/M)	Low (F/M)	Medium (F/M)	High (F/M)	Satellite	
					Control (F/M)	High (F/M)
Liver	4.41 ± 0.82/4.60 ± 0.30	4.09 ± 0.45/4.36 ± 0.15	4.77 ± 0.32/4.27 ± 0.38	4.23 ± 0.45/4.35 ± 0.17	5.01 ± 0.90/4.71 ± 1.08	4.52 ± 0.43/3.94 ± 0.26
Heart	0.51 ± 0.09/0.47 ± 0.01	0.45 ± 0.04/0.49 ± 0.06	0.49 ± 0.03/0.47 ± 0.03	0.44 ± 0.04^*^/0.45 ± 0.04	0.48 ± 0.06/0.41 ± 0.15	0.51 ± 0.07/0.45 ± 0.03
Spleen	0.36 ± 0.10/0.33 ± 0.05	0.38 ± 0.14/0.32 ± 0.04	0.36 ± 0.05/0.30 ± 0.03	0.32 ± 0.07/0.28 ± 0.09	0.41 ± 0.09/0.30 ± 0.07	0.30 ± 0.02^*^/0.26 ± 0.02
Lung	0.59 ± 0.05/0.55 ± 0.01	0.57 ± 0.04/0.51 ± 0.03	0.63 ± 0.04/0.56 ± 0.06	0.67 ± 0.07^*^/0.52 ± 0.02	0.55 ± 0.06/0.49 ± 0.06	0.58 ± 0.08/0.48 ± 0.07
Kidney (adrenal)	1.27 ± 0.12/1.70 ± 0.20	1.32 ± 0.06/1.71 ± 0.22	1.32 ± 0.08/1.79 ± 0.21	1.34 ± 0.20/1.65 ± 0.16	1.27 ± 0.06/1.69 ± 0.11	1.24 ± 0.03/1.60 ± 0.10
Thymus	0.28 ± 0.15/0.14 ± 0.06	0.29 ± 0.10/0.18 ± 0.07	0.28 ± 0.09/0.16 ± 0.07	0.25 ± 0.09/0.15 ± 0.01	0.18 ± 0.06/0.10 ± 0.03	0.19 ± 0.05/0.11 ± 0.04
Brain	1.08 ± 0.14/0.90 ± 0.10	1.12 ± 0.09/0.94 ± 0.11	1.22 ± 0.11/0.92 ± 0.12	1.14 ± 0.08/0.88 ± 0.14	1.01 ± 0.24/0.97 ± 0.12	1.54 ± 0.14^**^/1.11 ± 0.10
Bladder	0.05 ± 0.03/0.10 ± 0.04	0.04 ± 0.02/0.12 ± 0.04	0.05 ± 0.03/0.09 ± 0.06	0.08 ± 0.04/0.09 ± 0.02	0.04 ± 0.01/0.09 ± 0.04	0.05 ± 0.02/0.08 ± 0.02
Uterus (ovary)	0.87 ± 0.46/-	0.80 ± 0.16/-	0.61 ± 0.26/-	0.65 ± 0.05/-	0.62 ± 0.07/-	0.62 ± 0.15/-
Testis (epididymis)	-/1.18 ± 0.19	-/1.13 ± 0.16	-/1.12 ± 0.11	-/1.14 ± 0.19	-/0.88 ± 0.18	-/1.12 ± 0.14^*^
Seminal vesicle	-/0.38 ± 0.14	-/0.65 ± 0.11^**^	-/0.57 ± 0.08^*^	-/0.49 ± 0.10	-/0.48 ± 0.12	-/0.67 ± 0.17^*^

Note: Compared with sex-matched controls within each group, *: *P* < 0.05. * *: *P* < 0.01. F: female. M: male.

#### 3.4.5 HE staining pathological results

**FIGURE 3:**
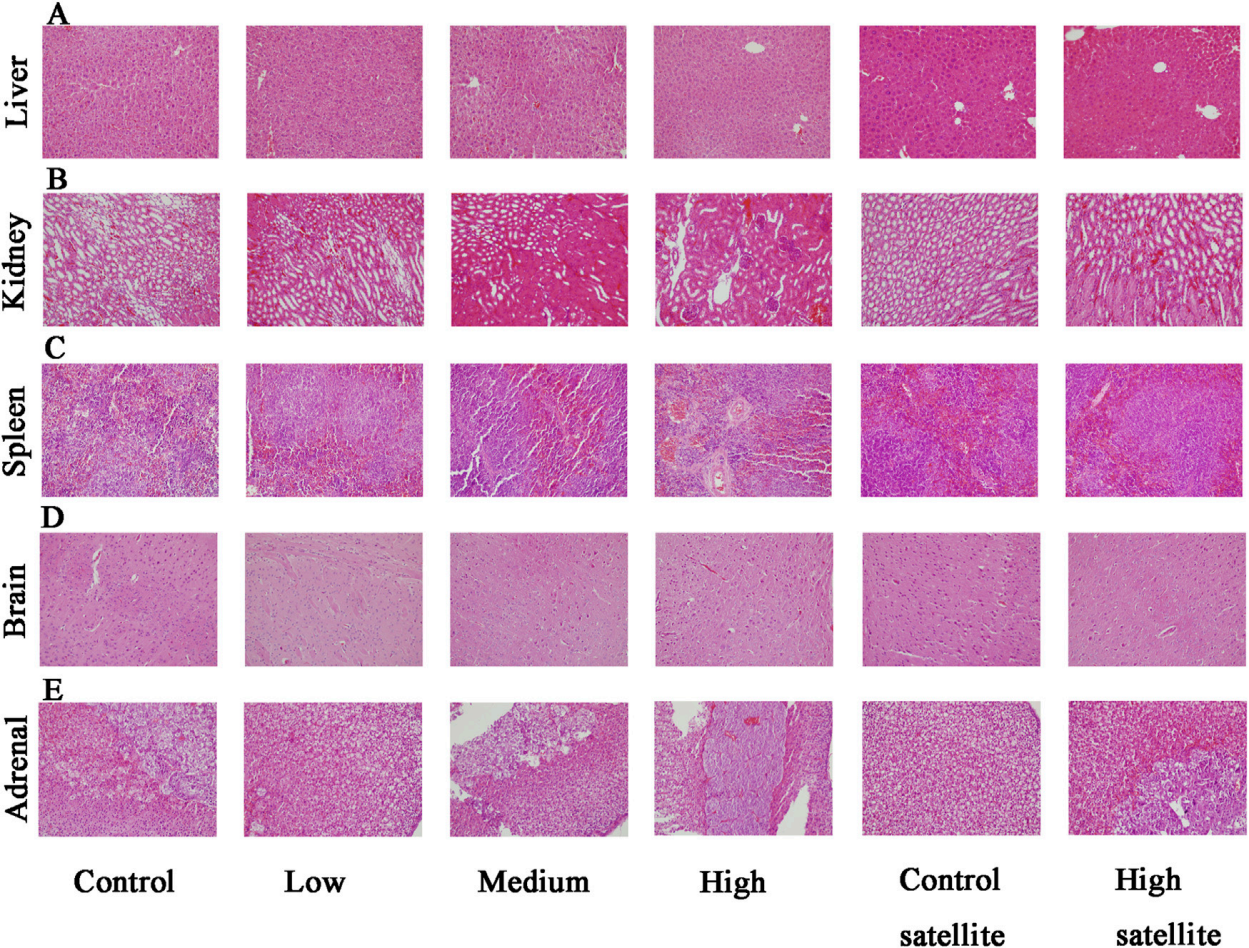
Histopathological findings of the liver, kidney, spleen, brain, and adrenal gland in mice following subacute toxicity testing. Note: **(A)** Liver exhibiting normal hepatic cords, sinusoids, deeply stained cytoplasm, and intact hepatocytes; no hydropic degeneration or laminar necrosis observed. **(B)** Kidney displaying structurally normal nephrons and collecting ducts without interstitial lipid deposition, inflammation, or mineralization. **(C)** Spleen demonstrating normal red and white pulp with no observed megakaryocytes or erythroid precursor cell clusters. **(D)** Brain tissue with orderly neuronal arrangement, no evidence of vacuolization, neuronal necrosis, inflammatory infiltration, or cellular debris. **(E)** Adrenal glands showing normal medullary and cortical regions, without proliferative foci characterized by eosinophilic cytoplasm.

**FIGURE 4:**
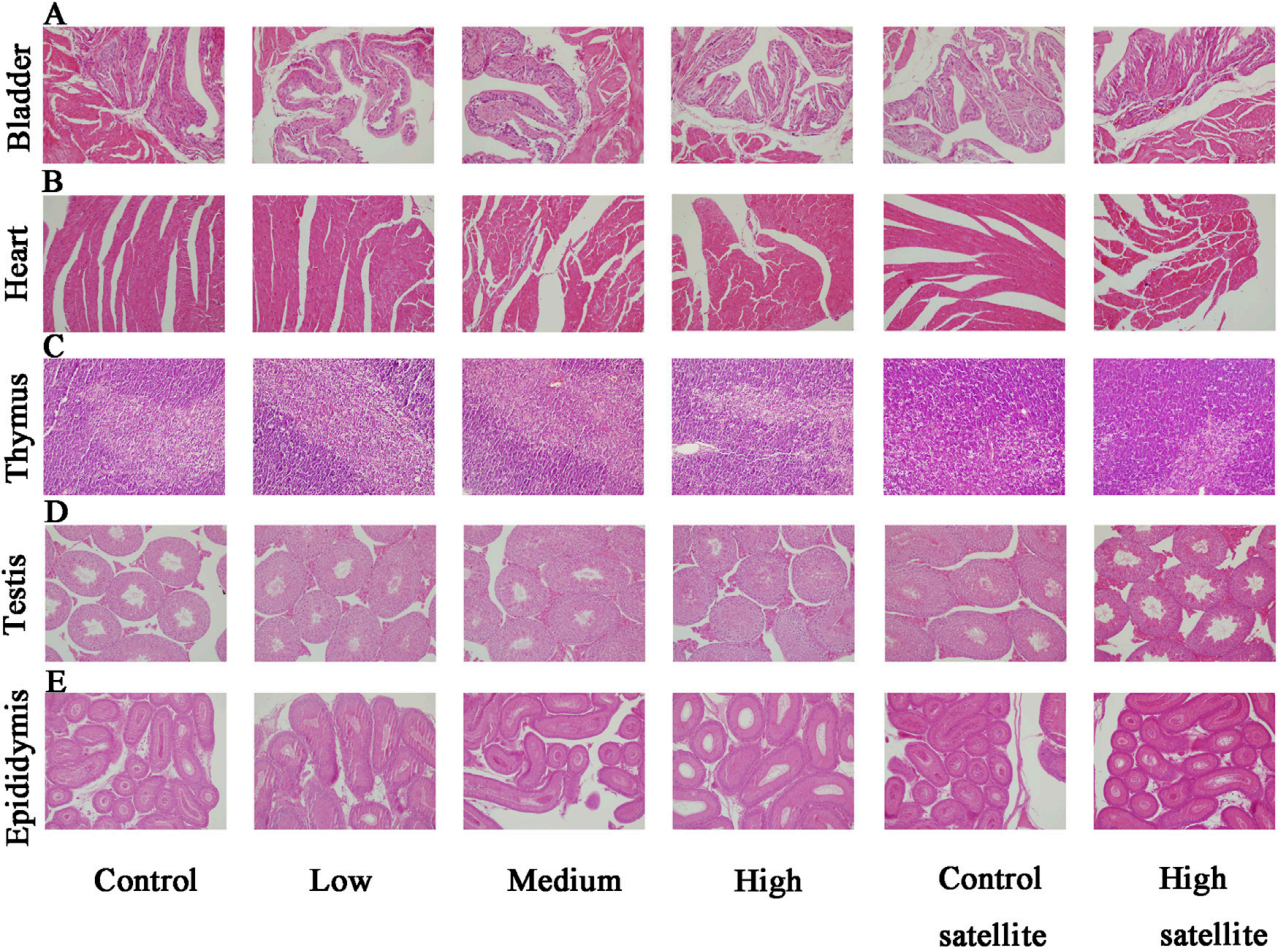
Histopathological findings of the bladder, heart, thymus, testis, and epididymis in mice following subacute toxicity testing. Note: **(A)** Bladder mucosa presenting multilayered folds with structurally normal umbrella cells; no multifocal lymphocyte or mast cell aggregates identified. **(B)** Heart sections displaying structurally intact myocardial fibers; no degenerative fragmentation, vacuolization, or increased eosinophil infiltration observed. **(C)** Thymus tissue clearly exhibiting distinct cortical and medullary regions. **(D)** Testicular tissue showing regular seminiferous tubules with normal spermatogenic and interstitial cells. **(E)** Epididymal ducts lined by morphologically normal epithelial cells with basally located nuclei.

**FIGURE 5:**
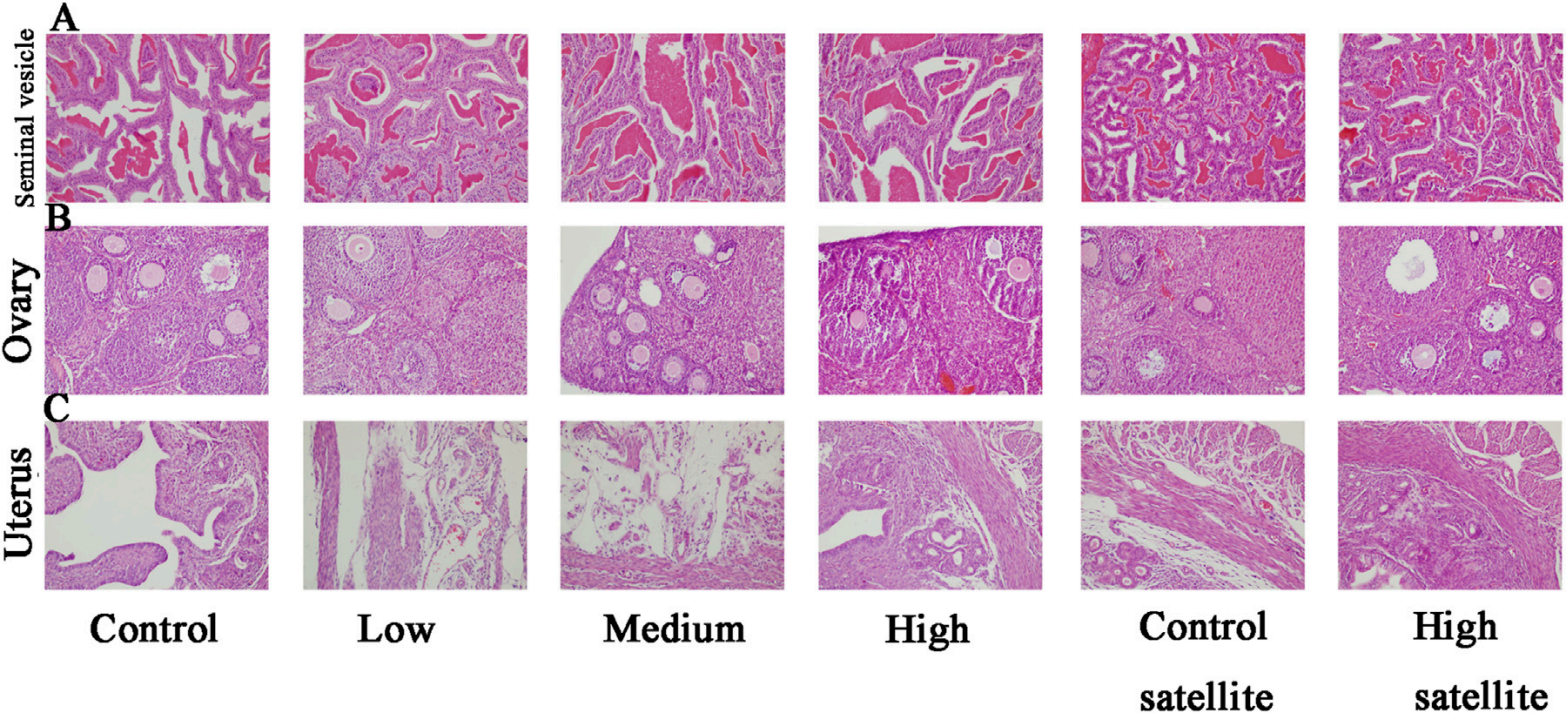
Histopathological findings of seminal vesicle, ovary, and uterus in mice following subacute toxicity testing. Note: **(A)** Seminal vesicles structurally normal, containing brightly eosinophilic secretions within the lumen. **(B)** Ovaries containing follicles at various stages of development, accompanied by normal stromal tissue. **(C)** Uterine tissue displaying normal epithelial cells and stromal structures, without evidence of extensive edema or increased neutrophil infiltration.

Histopathological analyses were performed on major organs, including the liver, kidneys, spleen, brain, adrenal glands, bladder, heart, thymus, testes, epididymides, seminal vesicles, ovaries, and uterus, across all experimental groups. The histopathological assessment revealed that the tissue structures in the treatment groups were comparable to those of the control group, without dose-dependent changes. Liver sections exhibited normal hepatic cords and sinusoids, deeply stained cytoplasm, and intact hepatocytes without hydropic degeneration or laminar necrosis (Figure 3A). Kidney sections displayed structurally normal nephrons and collecting ducts, with no evidence of interstitial lipid deposition, inflammation, or mineralization (Figure 3B). The spleen showed normal red and white pulp, with no observable megakaryocytes or clustered erythroid precursor cells (Figure 3C). Brain tissues demonstrated orderly neuronal arrangement, with no vacuolization, neuronal necrosis, inflammatory cell infiltration, or cellular debris detected (Figure 3D). The adrenal glands exhibited normal medullary and cortical regions without proliferative foci composed of eosinophilic cytoplasm (Figure 3E). Bladder mucosa showed multilayered folds and normal umbrella cells, free of multifocal lymphocyte or mast cell aggregations (Figure 4A). Heart sections revealed structurally intact myocardial fibers without signs of degenerative fragmentation, vacuolization, or eosinophil infiltration (Figure 4B). Thymus tissue displayed clearly defined cortical and medullary regions (Figure 4C). Testicular tissue exhibited regular seminiferous tubules containing normal spermatogenic and interstitial cells (Figure 4D). Epididymal ducts were lined by morphologically normal cells with basally positioned nuclei (Figure 4E). Seminal vesicles demonstrated normal structural integrity with brightly eosinophilic secretions filling the lumen (Figure 5A). Ovarian sections revealed follicles at various developmental stages and normal ovarian stroma (Figure 5B). The uterus displayed normal epithelial cells and stroma, with no evidence of widespread edema or neutrophil accumulation (Figure 5C).

## 4 Discussion

The clinical medicinal value and application of Traditional Chinese Medicine (TCM) are widely recognized for the treatment of various diseases, often serving as complementary or synergistic therapies alongside other treatment modalities (Huang et al., 2024; Feng et al., 2025). This versatility arises from the multi-molecular, multi-level, and multi-target nature of TCM, which enables flexible combinations of herbal formulations to enhance therapeutic efficacy while simultaneously addressing additional symptoms (Li et al., 2022). ZZXJD is a traditional formulation used clinically for the treatment of hepatocellular carcinoma; however, to date, no scientific data regarding its toxicity profile have been reported. Toxicological screening is a critical tool for evaluating drug safety, thus prompting acute and subacute toxicity studies of ZZXJD. Institute of Cancer Research (ICR) mice, characterized by stable peripheral blood profiles and commonly used for replicating pathological models, are widely employed in toxicological experiments (Ojiro et al., 2023; Ahn et al., 2025). Given that hematological analyses in rodents are among the most predictive indicators of human toxicity (Olson et al., 2000), ICR mice were selected as the primary experimental subjects for this study.

Initially, we conducted a preliminary *in vitro* toxicity assessment, in which the hepatocyte cell line HHL-5 and the renal cell line HEK-293 both demonstrated transient proliferation inhibition shortly after ZZXJD treatment. However, continued treatment reversed this inhibitory effect, indicating that the inhibitory action of ZZXJD on hepatic and renal cells was dose-dependent but not time-dependent. Further morphological experiments indicated that ZZXJD promoted apoptosis and necrosis in hepatic and renal cells within 24 h, likely explaining the observed inhibition of cell proliferation.

Acute toxicity testing enables rapid assessment of adverse drug reactions, providing valuable information for subsequent studies. In the present acute toxicity study, mice exhibited no mortality, adverse reactions, or behavioral abnormalities. Therefore, a single oral administration of ZZXJD at 10 g/kg was considered non-toxic.

Based on acute toxicity findings, a 28-day subacute toxicity experiment was conducted in male and female mice to evaluate potential cumulative organ toxicity and metabolic effects at low-dose exposures (Maru and Belemkar, 2025). Regarding body weight, all groups exhibited varying degrees of weight gain, which generally was not considered an adverse toxic reaction but rather attributed to normal physiological accumulation of body fat (El-Hajjaji et al., 2025). Food and water consumption remained stable without significant changes, indicating that ZZXJD did not negatively affect appetite or thirst and thus appeared not to disrupt the metabolic system of mice.

Relative organ weight is a critical toxicological parameter that reflects pathological changes such as congestion, edema, or degenerative lesions in affected organs (Bilal and Bibi, 2025). Except for seminal vesicles and testes, no significant differences in relative organ weights were found between treatment and control groups, particularly in the liver and kidneys, which are common targets of TCM-related toxicity. However, the observed variations in the relative weights of seminal vesicles and testes were not dose-dependent, and their increase or decrease was unrelated to treatment dosage, suggesting these changes might not result from organ-level toxicity induced by ZZXJD.

The hematopoietic system is a sensitive indicator commonly used for detecting toxic responses, reflecting physiological and pathological conditions within organisms (Suresh and Vellapandian, 2024). In this study, apart from lymphocytes and monocytes, no significant differences were observed in hematological parameters between treated and control groups. Although statistically significant elevations in lymphocytes at high doses and monocytes at intermediate doses were noted, these remained within the normal reference ranges for ICR mice and showed no dose-dependency, suggesting incidental occurrences (Patel et al., 2024). Thus, ZZXJD appears to have no harmful effects on the hematopoietic system and is unlikely to contain substances causing anemia or other chronic blood disorders.

Biochemical analyses indicated (Tourabi et al., 2024) more abnormalities in female mice compared to males, likely due to differences in pharmacokinetics and toxicokinetics influenced by factors such as average body size and hormonal status, rendering females more sensitive to adverse drug reactions (Gochfeld, 2017). Importantly, these biochemical abnormalities returned to normal 14 days after cessation of treatment, indicating that ZZXJD-induced biochemical changes were reversible. In terms of hepatic function, abnormal indicators predominantly appeared in females. Typically, elevations of AST and ALT levels serve as sensitive early markers of liver injury (Yuan et al., 2018); however, in this study, AST and ALT levels decreased to varying degrees, commonly interpreted as indicative of hepatic protection (Wang et al., 2023). Regarding renal function, significant reductions in BUN, CRE, and UA levels were observed in both sexes, an uncommon finding in clinical kidney injury assessments. Moreover, these changes lacked dose-dependency, implying they were likely incidental observations. Furthermore, considering the reference ranges for hepatic and renal parameters in ICR mice, some statistically significant alterations, such as ALT levels, remained within normal ranges (Patel et al., 2024). Due to the absence of additional abnormal indicators and inconsistencies between these findings and typical clinical assessments of hepatic and renal damage, ZZXJD was preliminarily regarded as non-toxic to the liver and kidneys.

Histopathological examinations supported findings from hematological and biochemical analyses (Liu et al., 2024). All tissues examined in control and treatment groups exhibited normal histological structures without dose-dependent damage. Specifically, no significant pathological changes were detected in the liver and kidneys after treatment, further suggesting that biochemical abnormalities were unrelated to toxic responses. Therefore, histopathological evidence supported the conclusion that ZZXJD was non-toxic.

Collectively, evidence from this study indicates that ZZXJD exhibited no toxic responses in acute or subacute toxicity assessments. In particular, the low dose in the subacute study, equivalent to the clinically recommended dosage, produced no abnormal hematological or biochemical findings. Although some changes occurred at medium and high doses (corresponding to twice and four times the clinical dose, respectively), these changes did not substantiate toxic effects and were reversible following cessation of treatment. Thus, ZZXJD is considered safe in acute and subacute toxicity studies and can be orally administered for at least 28 days without adverse effects.

Given that anticancer treatments often involve prolonged therapeutic regimens, further chronic toxicity studies are necessary. Additionally, evaluations of immunotoxicity, reproductive toxicity, and genotoxicity should be performed to ensure the safe clinical use of ZZXJD. These limitations will be addressed in future research.

## 5 Conclusion

The acute toxicity results of this study demonstrated that the LD50 of ZZXJD was greater than 10 g/kg in ICR mice, as no mortality occurred at this dosage. In the 28-day subacute toxicity study, ZZXJD produced no adverse effects on behavior, food and water intake, relative organ weights, hematological parameters, biochemical indices, or histopathology. However, certain indicators changed at doses exceeding clinical recommendations. Although these changes were not regarded as toxic, regular monitoring of hepatic and renal functions is recommended during clinical use as a precautionary measure.

## Data Availability

The original contributions presented in the study are included in the article/Supplementary Material, further inquiries can be directed to the corresponding author.
