# Supplementary material for: Acute and subacute toxicity evaluation of ZhenzhuXiaoji decoction in preclinical models: implications for safe clinical use
Source: Front Pharmacol. 2025 Apr 15;16:1554732. doi: 10.3389/fphar.2025.1554732 (PMC12037581; doi:10.3389/fphar.2025.1554732)
Supplement: Supplementary file 1 [file Table1.docx]

**Table S1 Bioactive constituents in the ZZXJD**

| **Number** | **name** | **mz** | **rt** | **exact_mass** | **ppm** | **formula** | **‰Relative content** |
| --- | --- | --- | --- | --- | --- | --- | --- |
| 1 | 2-Hydroxycinnamic acid | 165.0548 | 287.3 | 164.0473 | 1.211719 | C9H8O3 | 201.6335721 |
| 2 | Isoliquiritigenin | 257.0801 | 285.5 | 256.0736 | 3.018514 | C15H12O4 | 100.5976061 |
| 3 | alpha-Tocopherol | 430.3821 | 634.5 | 430.3811 | 2.323517 | C29H50O2 | 42.72153707 |
| 4 | L-Leucine | 132.1022 | 60.5 | 131.0946 | 0.552033 | C6H13NO2 | 33.91004612 |
| 5 | Quadrone | 231.1381 | 429.4 | 248.1412 | 18.75069 | C15H20O3 | 26.01968435 |
| 6 | Hydroxyphenyllactic acid | 165.0539 | 331.2 | 182.0579 | 20.80532 | C9H10O4 | 17.19185567 |
| 7 | 3-Hydroxybenzyl alcohol glucoside | 124.0877 | 643 | 124.0524 | 4.717873 | C7H8O2 | 16.7705578 |
| 8 | Confertifolin | 217.1603 | 511.8 | 234.162 | 26.40446 | C15H22O2 | 16.487189 |
| 9 | 4-Guanidinobutanoic acid | 146.0921 | 74.3 | 145.0851 | 1.889219 | C5H11N3O2 | 12.73880791 |
| 10 | Phenazine-1-carboxylate | 225.0757 | 287.3 | 224.0586 | 0.19816 | C13H8N2O2 | 9.756174255 |
| 11 | D-Arabitol | 151.0603 | 558.7 | 152.0685 | 0.023846 | C5H12O5 | 9.649088504 |
| 12 | 2-Furoate | 113.0234 | 89.6 | 112.016 | 1.097118 | C5H4O3 | 9.507026513 |
| 13 | L-Tyrosine | 182.0807 | 55.7 | 181.0739 | 2.614225 | C9H11NO3 | 9.460450498 |
| 14 | Gardenoside | 403.1232 | 126.7 | 404.1319 | 3.532419 | C17H24O11 | 9.457193906 |
| 15 | Alantolactone | 215.1432 | 448.7 | 232.1463 | 20.14472 | C15H20O2 | 8.269123386 |
| 16 | 13(S)-HpOTrE | 293.2117 | 455.7 | 310.2144 | 16.11311 | C18H30O4 | 7.946440603 |
| 17 | Caproic acid | 114.9343 | 624 | 116.0837 | 10.92421 | C6H12O2 | 7.920500973 |
| 18 | Eugenol | 165.0904 | 371.6 | 164.0837 | 2.453527 | C10H12O2 | 7.892949026 |
| 19 | Behenic acid | 339.3277 | 588.6 | 340.3341 | 2.491256 | C22H44O2 | 7.520634075 |
| 20 | 9(S)-HPOT | 293.2111 | 511.2 | 310.2144 | 0.036924 | C18H30O4 | 7.429096587 |
| 21 | Campesterol | 383.3692 | 672.5 | 400.3705 | 3.651832 | C28H48O | 7.398698426 |
| 22 | 5-(2-Hydroxyethyl)-4-methylthiazole | 144.047 | 91.9 | 143.0405 | 5.387131 | C6H9NOS | 7.055771761 |
| 23 | Adenine | 134.0473 | 58 | 135.0545 | 0.566964 | C5H5N5 | 6.918250022 |
| 24 | 4-Hydroxycinnamoylagmatine | 276.1445 | 76 | 276.1586 | 1.592922 | C14H20N4O2 | 6.561958648 |
| 25 | Metaraminol | 150.0911 | 86.7 | 167.0946 | 26.21075 | C9H13NO2 | 6.181914307 |
| 26 | Thiopental | 241.1081 | 190.6 | 242.1089 | 26.85932 | C11H18N2O2S | 6.171193939 |
| 27 | Uridine | 243.0611 | 78.1 | 244.0695 | 3.437098 | C9H12N2O6 | 6.169736282 |
| 28 | Coumarin | 147.0444 | 95.7 | 146.0368 | 0.477187 | C9H6O2 | 6.128105778 |
| 29 | Phenyllactate | 165.0554 | 136.9 | 166.063 | 1.962977 | C9H10O3 | 6.076117847 |
| 30 | Sorbitol | 165.0759 | 53.5 | 182.079 | 26.25459 | C6H14O6 | 5.937634326 |
| 31 | Estragole | 149.097 | 257.1 | 148.0888 | 3.748514 | C10H12O | 5.797293172 |
| 32 | O-Feruloylgalactarate | 387.1289 | 287.3 | 386.0849 | 1.044427 | C16H18O11 | 5.619615089 |
| 33 | Parthenolide | 249.1486 | 274.1 | 248.1412 | 0.497695 | C15H20O3 | 5.345238408 |
| 34 | 3,4-Dihydroxyphenylglycol | 169.0519 | 78.1 | 170.0579 | 7.547978 | C8H10O4 | 5.315035634 |
| 35 | Myristicin | 193.0859 | 339.8 | 192.0786 | 0.124297 | C11H12O3 | 5.265249615 |
| 36 | Histamine | 112.0874 | 55.7 | 111.0796 | 0.02091 | C5H9N3 | 5.261653993 |
| 37 | Linoleic acid | 280.2639 | 586.8 | 280.2402 | 3.825681 | C18H32O2 | 5.161791369 |
| 38 | Aminoadipic acid | 144.0652 | 52.6 | 161.0688 | 26.61295 | C6H11NO4 | 5.158219122 |
| 39 | 4-Methylcatechol | 123.0448 | 76.9 | 124.0524 | 2.633187 | C7H8O2 | 5.146837969 |
| 40 | Sinapoyl aldehyde | 209.0798 | 328.2 | 208.0736 | 4.111613 | C11H12O4 | 5.094007815 |
| 41 | 3-Hydroxymethylglutaric acid | 144.9826 | 675.6 | 162.0528 | 1.86007 | C6H10O5 | 4.830791212 |
| 42 | L-Carnitine | 162.1121 | 52.5 | 161.1052 | 1.535817 | C7H15NO3 | 4.581721401 |
| 43 | 2-trans,6-trans-Farnesal | 221.1901 | 483.6 | 220.1827 | 0.4521 | C15H24O | 4.545151215 |
| 44 | 2-Dehydropantoate | 146.06 | 324.6 | 146.0579 | 0.001387 | C6H10O4 | 4.491045897 |
| 45 | Piperonal | 151.04 | 320.8 | 150.0317 | 4.938395 | C8H6O3 | 4.240227958 |
| 46 | Neocnidilide | 195.1384 | 332.4 | 194.1307 | 0.87533 | C12H18O2 | 4.12795625 |
| 47 | Phthalic acid | 149.0232 | 551.5 | 166.0266 | 27.06961 | C8H6O4 | 3.917181452 |
| 48 | Parthenin | 263.1282 | 406.3 | 262.1205 | 1.143283 | C15H18O4 | 3.916456349 |
| 49 | L-2-Hydroxyglutaric acid | 149.0244 | 234.6 | 148.0372 | 5.741935 | C5H8O5 | 3.884269428 |
| 50 | Sinapic acid | 207.0654 | 298.2 | 224.0685 | 20.93059 | C11H12O5 | 3.704883726 |
| 51 | Dihydrouracil | 115.0397 | 104 | 114.0429 | 2.822459 | C4H6N2O2 | 3.625664769 |
| 52 | alpha-Santonin | 229.1228 | 246.3 | 246.1256 | 20.22496 | C15H18O3 | 3.560980814 |
| 53 | 4-Imidazolone-5-propionic acid | 156.0423 | 50.4 | 156.0535 | 0.298683 | C6H8N2O3 | 3.560952025 |
| 54 | Cyclic AMP | 330.0595 | 55.7 | 329.0525 | 0.836213 | C10H12N5O6P | 3.554177843 |
| 55 | Guanosine | 282.0846 | 78.1 | 283.0917 | 0.623926 | C10H13N5O5 | 3.548475509 |
| 56 | Stearic acid | 283.2617 | 603.8 | 284.2715 | 8.910488 | C18H36O2 | 3.519502153 |
| 57 | Safrole | 163.0766 | 456.7 | 162.0681 | 7.358505 | C10H10O2 | 3.473563869 |
| 58 | 2,3-Butanediol | 154.9901 | 508.2 | 154.0122 | 0.506536 | C4H10O2S2 | 3.375659995 |
| 59 | Phenylacetic acid | 137.0595 | 95.7 | 136.0524 | 0.317944 | C8H8O2 | 3.352414466 |
| 60 | 2-Methylserine | 119.0497 | 75.4 | 119.0582 | 0.014201 | C4H9NO3 | 3.276804988 |
| 61 | Cyanazine | 240.0869 | 55.7 | 240.089 | 8.746833 | C9H13ClN6 | 3.185374298 |
| 62 | Erythritol | 121.0295 | 80.5 | 122.0579 | 8.998598 | C4H10O4 | 3.101252123 |
| 63 | 4-Hydroxybenzoic acid | 137.0245 | 451.1 | 138.0317 | 0.554645 | C7H6O3 | 3.081918987 |
| 64 | Diglycidyl resorcinol ether | 205.0851 | 267.9 | 222.0892 | 16.25667 | C12H14O4 | 2.915228899 |
| 65 | Medicarpin | 271.0968 | 427.9 | 270.0892 | 1.195145 | C16H14O4 | 2.828600388 |
| 66 | D-Mannose | 179.0576 | 192 | 180.0634 | 8.243158 | C6H12O6 | 2.810343447 |
| 67 | Riboflavin | 377.1454 | 253.3 | 376.1383 | 0.463629 | C17H20N4O6 | 2.792110716 |
| 68 | Isoscopoletin | 193.0499 | 228.3 | 192.0423 | 0.795441 | C10H8O4 | 2.75499131 |
| 69 | Pantothenic acid | 218.1037 | 78.1 | 219.1107 | 1.265453 | C9H17NO5 | 2.753812256 |
| 70 | Cantharidin | 197.0808 | 297.5 | 196.0736 | 0.385629 | C10H12O4 | 2.726237947 |
| 71 | Capsidiol | 219.1746 | 253.3 | 236.1776 | 1.368772 | C15H24O2 | 2.696336635 |
| 72 | 3-Hydroxyflavone | 237.0554 | 557.2 | 238.063 | 1.342218 | C15H10O3 | 2.622077454 |
| 73 | Dulcin | 181.0967 | 90.8 | 180.0899 | 1.857382 | C9H12N2O2 | 2.52926581 |
| 74 | D-Lyxose | 150.054 | 248.8 | 150.0528 | 7.997121 | C5H10O5 | 2.487575512 |
| 75 | Acetosyringone | 195.0664 | 254.7 | 196.0736 | 0.389611 | C10H12O4 | 2.486428188 |
| 76 | 3D-3,5_4-Trihydroxycyclohexane-1,2-dione | 161.0609 | 297.1 | 160.0372 | 6.484741 | C6H8O5 | 2.456932397 |
| 77 | Ketoleucine | 131.0855 | 440.5 | 130.063 | 27.00495 | C6H10O3 | 2.45402604 |
| 78 | (R)-Higenamine | 272.1284 | 238.5 | 271.1208 | 0.036618 | C16H17NO3 | 2.412071956 |
| 79 | (1S,4R)-1-Hydroxy-2-oxolimonene | 169.1222 | 306 | 168.115 | 0.449379 | C10H16O2 | 2.405899998 |
| 80 | 3-Methylthiopropionic acid | 119.0496 | 497.8 | 120.0245 | 0.780002 | C4H8O2S | 2.381512139 |
| 81 | 9,10-EOT | 293.2116 | 579.3 | 292.2038 | 1.787108 | C18H28O3 | 2.350505945 |
| 82 | D-Galactose | 179.0544 | 162 | 180.0634 | 9.628266 | C6H12O6 | 2.32814694 |
| 83 | L-Isoleucine | 130.0871 | 58 | 131.0946 | 1.721923 | C6H13NO2 | 2.317756427 |
| 84 | Succinic acid | 117.019 | 408 | 118.0266 | 2.768781 | C4H6O4 | 2.302644634 |
| 85 | (Z)-4-Hydroxy-6-dodecenoic acid lactone | 179.0704 | 271.5 | 178.0477 | 0.829479 | C6H10O6 | 2.277755493 |
| 86 | Norepinephrine | 169.0762 | 259.1 | 169.0739 | 13.60333 | C8H11NO3 | 2.270720277 |
| 87 | Prostaglandin F3a | 353.2317 | 363.4 | 352.225 | 1.630658 | C20H32O5 | 2.230707267 |
| 88 | Hispidulin | 299.0559 | 342.8 | 300.0634 | 0.749024 | C16H12O6 | 2.194842928 |
| 89 | Ferreirin | 303.0859 | 300.7 | 302.079 | 0.399285 | C16H14O6 | 2.17408082 |
| 90 | 4-(beta-D-Glucosyloxy)benzoate | 299.0761 | 164.6 | 300.0845 | 3.758227 | C13H16O8 | 2.141793302 |
| 91 | Nicotinic acid | 122.0252 | 56.8 | 123.032 | 3.900834 | C6H5NO2 | 2.125716823 |
| 92 | Methyleugenol | 179.1077 | 260.2 | 178.0994 | 5.717263 | C11H14O2 | 2.075939554 |
| 93 | Daidzein | 255.0655 | 400.2 | 254.0579 | 1.176168 | C15H10O4 | 2.0726778 |
| 94 | Hydroquinone | 110.0206 | 682.5 | 110.0368 | 0.075115 | C6H6O2 | 2.069109463 |
| 95 | L-Asparagine | 133.061 | 52.5 | 132.0535 | 1.683438 | C4H8N2O3 | 2.062158306 |
| 96 | Alpha-D-Glucose | 161.0446 | 505.6 | 180.0634 | 2.545879 | C6H12O6 | 2.046750032 |
| 97 | Jasmone | 165.1277 | 461 | 164.1201 | 1.962118 | C11H16O | 2.001792467 |
| 98 | (R)-Glabridin | 323.1288 | 475.1 | 324.1362 | 0.257235 | C20H20O4 | 1.998553758 |
| 99 | L-Phenylalanine | 166.0858 | 327.3 | 165.079 | 2.865989 | C9H11NO2 | 1.993273233 |
| 100 | p-Anisic acid | 151.0391 | 354.3 | 152.0473 | 6.117621 | C8H8O3 | 1.989746791 |
| 101 | N6-Acetyl-L-lysine | 189.1246 | 74.6 | 188.1161 | 3.209948 | C8H16N2O3 | 1.968694741 |
| 102 | 4-Hydroxycinnamic acid | 163.0387 | 320.7 | 164.0473 | 2.685744 | C9H8O3 | 1.962630384 |
| 103 | Genistein | 269.0455 | 427.1 | 270.0528 | 0.089204 | C15H10O5 | 1.925025557 |
| 104 | Azelaic acid | 187.0961 | 473.5 | 188.1049 | 0.679572 | C9H16O4 | 1.924561985 |
| 105 | Tyrosol | 121.0657 | 421.3 | 138.0681 | 5.320688 | C8H10O2 | 1.917039617 |
| 106 | Geranyl diphosphate | 314.0893 | 75.4 | 314.0684 | 0.241969 | C10H20O7P2 | 1.901214206 |
| 107 | Naringenin | 273.076 | 372.9 | 272.0685 | 0.820284 | C15H12O5 | 1.82981319 |
| 108 | Chlorogenic acid | 355.1027 | 231 | 354.0951 | 0.004889 | C16H18O9 | 1.825396574 |
| 109 | Indolebutyric acid | 204.103 | 568.6 | 203.0946 | 5.507023 | C12H13NO2 | 1.821635769 |
| 110 | Guanine | 151.0401 | 117.5 | 151.0494 | 6.816972 | C5H5N5O | 1.815457788 |
| 111 | Neochlorogenic acid | 353.0868 | 58 | 354.0951 | 2.900137 | C16H18O9 | 1.786497198 |
| 112 | Lycopene | 536.4377 | 614.6 | 536.4382 | 0.520228 | C40H56 | 1.726818075 |
| 113 | Pyroglutamic acid | 128.0358 | 94.2 | 129.0426 | 3.71771 | C5H7NO3 | 1.707945223 |
| 114 | Glycylleucine | 189.1234 | 136 | 188.1161 | 0.126901 | C8H16N2O3 | 1.690836156 |
| 115 | (R)-5,6-Dihydrothymine | 128.0617 | 363.4 | 128.0586 | 24.20708 | C5H8N2O2 | 1.645354831 |
| 116 | 3-Hydroxyvalproic acid | 159.1025 | 260.3 | 160.1099 | 0.779372 | C8H16O3 | 1.637281478 |
| 117 | Caryophyllene alpha-oxide | 203.1796 | 622.4 | 220.1827 | 21.33088 | C15H24O | 1.631230291 |
| 118 | 1,2,3-Trihydroxybenzene | 127.0385 | 287.3 | 126.0317 | 3.746896 | C6H6O3 | 1.630369991 |
| 119 | Andrographolide | 333.2052 | 440.5 | 350.2093 | 10.00585 | C20H30O5 | 1.630276604 |
| 120 | Methyl jasmonate | 225.1483 | 248.2 | 224.1412 | 0.394806 | C13H20O3 | 1.621521804 |
| 121 | 1,2-Epoxy-p-menth-8-ene | 153.1276 | 254.7 | 152.1201 | 1.3061 | C10H16O | 1.565318916 |
| 122 | 2-Phenylacetamide | 136.0766 | 265.3 | 135.0684 | 6.688086 | C8H9NO | 1.559357325 |
| 123 | N(6)-Methyllysine | 161.1319 | 496.7 | 160.1212 | 3.302803 | C7H16N2O2 | 1.557683739 |
| 124 | Vitexin | 433.1124 | 281.2 | 432.1056 | 1.099022 | C21H20O10 | 1.550387016 |
| 125 | Genipin | 227.0913 | 294.5 | 226.0841 | 0.334667 | C11H14O5 | 1.548836432 |
| 126 | 1,3-Benzenediol | 109.0291 | 242.7 | 110.0368 | 3.668745 | C6H6O2 | 1.53267328 |
| 127 | m-Cresol | 109.1014 | 315.6 | 108.0575 | 0.580358 | C7H8O | 1.529253365 |
| 128 | DHHA | 155.0711 | 91.9 | 155.0582 | 5.803611 | C7H9NO3 | 1.50242402 |
| 129 | Uric acid | 169.0486 | 227 | 168.0283 | 9.300332 | C5H4N4O3 | 1.500149561 |
| 130 | Diethylpropion | 206.1543 | 218.6 | 205.1467 | 1.571638 | C13H19NO | 1.473075817 |
| 131 | L-Histidinol | 141.0907 | 265.3 | 141.0902 | 1.86933 | C6H11N3O | 1.471588068 |
| 132 | Perillyl alcohol | 153.0903 | 385.9 | 152.1201 | 4.981857 | C10H16O | 1.416522673 |
| 133 | D-Xylitol | 151.0624 | 381.6 | 152.0685 | 7.784862 | C5H12O5 | 1.41580717 |
| 134 | Cytidine | 244.0931 | 53.5 | 243.0855 | 0.025105 | C9H13N3O5 | 1.410236314 |
| 135 | Nandrolone | 257.19 | 440.1 | 274.1933 | 16.07372 | C18H26O2 | 1.403237457 |
| 136 | Perillyl aldehyde | 150.0995 | 472.2 | 150.1045 | 0.42371 | C10H14O | 1.397826069 |
| 137 | 3-Hydroxyphenylacetic acid | 153.0545 | 355.1 | 152.0473 | 0.496555 | C8H8O3 | 1.392908439 |
| 138 | Adipate semialdehyde | 130.0496 | 455.7 | 130.063 | 3.639463 | C6H10O3 | 1.359679401 |
| 139 | Trenbolone | 271.1689 | 438.8 | 270.162 | 1.38659 | C18H22O2 | 1.34113418 |
| 140 | Xanthoxin | 251.1646 | 265.3 | 250.1569 | 0.009321 | C15H22O3 | 1.324834247 |
| 141 | Deacetylipecoside | 523.1797 | 275 | 523.2054 | 1.814395 | C25H33NO11 | 1.316646869 |
| 142 | trans-Ferulic acid | 193.0494 | 458.6 | 194.0579 | 2.412453 | C10H10O4 | 1.311926312 |
| 143 | Jasmonic acid | 193.1227 | 248.8 | 210.1256 | 23.4773 | C12H18O3 | 1.311426943 |
| 144 | 3-Methylindole | 132.081 | 472.2 | 131.0735 | 1.514222 | C9H9N | 1.300108029 |
| 145 | Butabarbital | 213.1274 | 468 | 212.1161 | 18.76812 | C10H16N2O3 | 1.25995585 |
| 146 | (2R,5S)-2,5-Diaminohexanoate | 146.1175 | 119.3 | 146.1055 | 0.364473 | C6H14N2O2 | 1.251616867 |
| 147 | N,N-Dimethylhistidine | 183.1029 | 329.4 | 183.1008 | 6.207746 | C8H13N3O2 | 1.238918145 |
| 148 | Fenfluramine | 232.1329 | 249.9 | 231.1235 | 0.950582 | C12H16F3N | 1.219324694 |
| 149 | Rosmarinate | 361.0927 | 311.7 | 360.0845 | 2.5589 | C18H16O8 | 1.21439372 |
| 150 | L-Glutamic acid | 128.0348 | 295.2 | 147.0532 | 0.078104 | C5H9NO4 | 1.189380619 |
| 151 | Gamma-Linolenic acid | 277.2174 | 499.6 | 278.2246 | 0.274153 | C18H30O2 | 1.186674074 |
| 152 | Androsterone | 291.1955 | 291.4 | 290.2246 | 2.604934 | C19H30O2 | 1.174783736 |
| 153 | Gallic acid | 171.0291 | 205.6 | 170.0215 | 0.008708 | C7H6O5 | 1.16241604 |
| 154 | (S)-Abscisic acid | 265.1433 | 278.9 | 264.1362 | 0.377155 | C15H20O4 | 1.137562455 |
| 155 | trans-Cinnamate | 131.0493 | 471.1 | 148.0524 | 0.10768 | C9H8O2 | 1.135542186 |
| 156 | (2S)-Liquiritigenin | 257.0815 | 477.7 | 256.0736 | 0.976942 | C15H12O4 | 1.126599374 |
| 157 | o-Xylene | 107.0858 | 255.8 | 106.0783 | 2.091781 | C8H10 | 1.121686628 |
| 158 | 6-Methylmercaptopurine | 166.0501 | 239.9 | 166.0313 | 12.18909 | C6H6N4S | 1.109531784 |
| 159 | Methylmalonic acid | 117.0195 | 509.2 | 118.0266 | 1.504023 | C4H6O4 | 1.097711067 |
| 160 | Formononetin | 269.0781 | 489.4 | 268.0736 | 10.31671 | C16H12O4 | 1.064197872 |
| 161 | 2-Phenylethanol | 105.0701 | 378 | 122.0732 | 0.025404 | C8H10O | 1.041034483 |
| 162 | Bovinic acid | 279.2355 | 668.7 | 280.2402 | 8.953016 | C18H32O2 | 1.03470155 |
| 163 | 3,4-Dihydroxybenzaldehyde | 137.0237 | 678.1 | 138.0317 | 5.108605 | C7H6O3 | 0.994987805 |
| 164 | 13S-hydroxyoctadecadienoic acid | 296.2303 | 477.7 | 296.2351 | 16.20335 | C18H32O3 | 0.98497119 |
| 165 | Phosphoribosylformylglycineamidine | 313.0722 | 316 | 313.0675 | 11.61247 | C8H16N3O8P | 0.972493646 |
| 166 | D-Gulono-1,4-lactone | 178.0499 | 150 | 178.048 | 10.67117 | C6H10O6 | 0.9693836 |
| 167 | Arachidonic acid | 305.248 | 333.3 | 304.2402 | 0.033927 | C20H32O2 | 0.960806622 |
| 168 | Diaminopimelic acid | 190.1076 | 54.6 | 190.0954 | 0.26699 | C7H14N2O4 | 0.960384749 |
| 169 | Myclobutanil | 289.1172 | 234.6 | 288.1142 | 2.595624 | C15H17ClN4 | 0.952316291 |
| 170 | Hordenine | 166.1228 | 76 | 165.1154 | 0.746436 | C10H15NO | 0.941103141 |
| 171 | L-Erythrulose | 101.0241 | 192 | 120.0423 | 1.880739 | C4H8O4 | 0.938542823 |
| 172 | Xanthoxylin | 195.0664 | 294.4 | 196.0736 | 0.389611 | C10H12O4 | 0.938063359 |
| 173 | Ribitol | 151.0624 | 644.6 | 152.0685 | 7.784862 | C5H12O5 | 0.928818843 |
| 174 | Fisetin | 287.055 | 346.3 | 286.048 | 0.961488 | C15H10O6 | 0.917452397 |
| 175 | 12,13-DHOME | 315.2539 | 375.4 | 314.2457 | 2.930971 | C18H34O4 | 0.914383392 |
| 176 | p-Synephrine | 168.1021 | 324.2 | 167.0946 | 1.189753 | C9H13NO2 | 0.900219783 |
| 177 | L-Glutamine | 146.0727 | 472.4 | 146.0691 | 24.64526 | C5H10N2O3 | 0.898239514 |
| 178 | N-Alpha-acetyllysine | 171.1126 | 210 | 188.1161 | 22.99071 | C8H16N2O3 | 0.897779029 |
| 179 | Tetrahydropteridine | 136.0758 | 305.6 | 136.0749 | 6.613961 | C6H8N4 | 0.897458933 |
| 180 | Melibiitol | 325.1145 | 54.3 | 344.1319 | 3.04509 | C12H24O11 | 0.891217585 |
| 181 | 1-Hexadecanol | 243.1834 | 186.7 | 242.261 | 4.285703 | C16H34O | 0.889544006 |
| 182 | Practolol | 265.1479 | 627 | 266.163 | 29.50806 | C14H22N2O3 | 0.886159836 |
| 183 | Luteolin | 285.0403 | 495 | 286.0477 | 0.435026 | C15H10O6 | 0.87382401 |
| 184 | trans-1,2-Cyclohexanediol | 115.9211 | 682.6 | 116.0837 | 11.94018 | C6H12O2 | 0.853642145 |
| 185 | Ergosta-5,7,22,24(28)-tetraen-3beta-ol | 377.3221 | 622.1 | 394.3236 | 15.72662 | C28H42O | 0.84519545 |
| 186 | Cortisol | 361.2025 | 507.1 | 362.2093 | 1.31782 | C21H30O5 | 0.844233596 |
| 187 | Vanillic acid | 167.0351 | 272.1 | 168.0423 | 0.454994 | C8H8O4 | 0.821045004 |
| 188 | Versicolorone | 387.1438 | 313.7 | 386.1002 | 0.043226 | C20H18O8 | 0.817824714 |
| 189 | Arbutin | 271.0829 | 78.1 | 272.0896 | 2.124811 | C12H16O7 | 0.786094411 |
| 190 | Punicic acid | 278.2195 | 364.7 | 278.2246 | 18.33051 | C18H30O2 | 0.766998791 |
| 191 | Apigenin | 269.0428 | 620.5 | 270.0528 | 9.300533 | C15H10O5 | 0.756167209 |
| 192 | 3-Amino-4-hydroxybenzoate | 152.08 | 157.5 | 153.0426 | 27.90488 | C7H7NO3 | 0.732801567 |
| 193 | Cyanidin 3-glucoside | 449.1042 | 284.8 | 449.1084 | 7.591662 | C21H21O11 | 0.696689976 |
| 194 | Suberic acid | 173.0821 | 91.8 | 174.0892 | 1.016858 | C8H14O4 | 0.696061596 |
| 195 | Abscisic alcohol | 251.1676 | 512.6 | 250.1569 | 11.55393 | C15H22O3 | 0.689502698 |
| 196 | Benzaldehyde | 107.0497 | 76.5 | 106.0419 | 0.019841 | C7H6O | 0.666156274 |
| 197 | all-trans-Retinoic acid | 301.2155 | 527.3 | 300.2089 | 2.24424 | C20H28O2 | 0.637678674 |
| 198 | Succinic acid semialdehyde | 101.024 | 410.2 | 102.0317 | 4.197005 | C4H6O3 | 0.627470763 |
| 199 | p-Cresol | 109.0652 | 181.6 | 108.0575 | 3.887598 | C7H8O | 0.618363488 |
| 200 | 3,4-Methylenedioxyamphetamine | 194.1179 | 627.6 | 193.1103 | 0.063325 | C11H15NO2 | 0.617727706 |
| 201 | Prephenate | 207.03 | 110.1 | 226.0477 | 3.332861 | C10H10O6 | 0.612404632 |
| 202 | Alpha-Linolenic acid | 261.2217 | 501.2 | 278.2246 | 17.3569 | C18H30O2 | 0.584717988 |
| 203 | Stearolic acid | 281.2477 | 464.5 | 280.2402 | 0.796451 | C18H32O2 | 0.560433199 |
| 204 | Vanillylmandelic acid | 197.119 | 421.5 | 198.0528 | 6.098592 | C9H10O5 | 0.544609508 |
| 205 | Dihydrochelirubine | 363.1078 | 303.1 | 363.1107 | 7.986609 | C21H17NO5 | 0.543203985 |
| 206 | Propionylcarnitine | 218.1383 | 83.5 | 217.1314 | 1.723677 | C10H19NO4 | 0.534734207 |
| 207 | Misoprostol | 369.264 | 419.9 | 368.2563 | 1.217931 | C21H36O5 | 0.530529157 |
| 208 | Phenyl acetate | 135.9704 | 667.1 | 136.0524 | 5.513931 | C8H8O2 | 0.528648566 |
| 209 | Shikimic acid | 173.0455 | 275.8 | 174.0528 | 0 | C7H10O5 | 0.519758849 |
| 210 | Dodecanedioic acid | 211.1349 | 419.4 | 230.1518 | 7.0571 | C12H22O4 | 0.515392904 |
| 211 | Secologanin | 389.1433 | 220.7 | 388.1369 | 2.251094 | C17H24O10 | 0.512412771 |
| 212 | Maslinic acid | 455.3523 | 607.7 | 472.3553 | 9.737515 | C30H48O4 | 0.508630691 |
| 213 | Linalool | 155.1431 | 260.3 | 154.1358 | 0.154696 | C10H18O | 0.506217734 |
| 214 | Pelargonic acid | 141.1275 | 376.2 | 158.1307 | 0.214883 | C9H18O2 | 0.503109399 |
| 215 | Flavonol 7-O-beta-D-glucoside | 417.1221 | 351.6 | 416.1107 | 9.886793 | C21H20O9 | 0.49562191 |
| 216 | Methylisoeugenol | 179.1073 | 501.7 | 178.0994 | 3.483945 | C11H14O2 | 0.492471589 |
| 217 | Qing Hau Sau | 283.1539 | 377.1 | 282.1467 | 0.268405 | C15H22O5 | 0.486797187 |
| 218 | 3-Methoxyanthranilate | 166.051 | 406.7 | 167.0582 | 0 | C8H9NO3 | 0.486762061 |
| 219 | Pregnanediol | 321.3151 | 630 | 320.2715 | 0.01857 | C21H36O2 | 0.475978837 |
| 220 | Juvenile hormone III | 249.185 | 528.5 | 266.1882 | 16.99139 | C16H26O3 | 0.4742458 |
| 221 | Glycyrrhetinic acid | 469.3329 | 482.3 | 470.3396 | 1.206069 | C30H46O4 | 0.471375448 |
| 222 | N-Succinyl-2-amino-6-ketopimelate | 289.0708 | 257.1 | 289.0798 | 0.124608 | C11H15NO8 | 0.458345471 |
| 223 | Prostaglandin F1a | 355.2494 | 484.4 | 356.2563 | 1.058411 | C20H36O5 | 0.441491713 |
| 224 | Erucic acid | 337.2081 | 501 | 338.3185 | 7.965946 | C22H42O2 | 0.440745006 |
| 225 | Stearidonic acid | 259.2056 | 477.7 | 276.2089 | 15.94873 | C18H28O2 | 0.428813868 |
| 226 | Pimelate | 141.0558 | 47.7 | 160.0736 | 4.182742 | C7H12O4 | 0.413748824 |
| 227 | 9-OxoODE | 295.2267 | 558.2 | 294.2195 | 0.257429 | C18H30O3 | 0.412112709 |
| 228 | 5-Carboxyvanillic acid | 211.0248 | 254.7 | 212.0321 | 0.113731 | C9H8O6 | 0.405486683 |
| 229 | Cholesterol sulfate | 465.304 | 578.1 | 466.3117 | 0.859653 | C27H46O4S | 0.391036834 |
| 230 | 1-Keto-D-chiro-inositol | 179.034 | 400.6 | 178.0477 | 17.58058 | C6H10O6 | 0.390695881 |
| 231 | 9-cis-Retinoic acid | 301.2157 | 475.4 | 300.2089 | 1.580263 | C20H28O2 | 0.38379414 |
| 232 | Thymidine | 223.0282 | 640.8 | 242.0903 | 1.379275 | C10H14N2O5 | 0.381881464 |
| 233 | Betaine | 118.065 | 641.3 | 117.079 | 25.6982 | C5H11NO2 | 0.363725481 |
| 234 | Arabidiol | 445.3701 | 586.8 | 444.3967 | 3.633735 | C30H52O2 | 0.359078734 |
| 235 | Oleanolic acid | 457.3692 | 552.5 | 456.3603 | 3.550742 | C30H48O3 | 0.353311325 |
| 236 | Hexadecanedioate | 267.1977 | 391.1 | 286.2144 | 6.324905 | C16H30O4 | 0.346576627 |
| 237 | Schaftoside | 565.165 | 251.3 | 564.1479 | 17.38253 | C26H28O14 | 0.344635794 |
| 238 | Daidzin | 397.2239 | 478.3 | 416.1107 | 6.473807 | C21H20O9 | 0.337750414 |
| 239 | Dehydroepiandrosterone | 289.2157 | 534 | 288.2089 | 1.64583 | C19H28O2 | 0.326001271 |
| 240 | Desmethylxanthohumol | 339.1238 | 438.7 | 340.1311 | 0.006664 | C20H20O5 | 0.318658743 |
| 241 | Palmitoylethanolamide | 300.2899 | 591.3 | 299.2824 | 0.666023 | C18H37NO2 | 0.304031185 |
| 242 | Indoleacetaldehyde | 160.0719 | 71.9 | 159.0684 | 22.68359 | C10H9NO | 0.301539707 |
| 243 | Creatinine | 113.0609 | 76.7 | 113.0589 | 17.68958 | C4H7N3O | 0.293499435 |
| 244 | 8-Amino-7-oxononanoate | 170.1177 | 561.2 | 187.1208 | 25.47648 | C9H17NO3 | 0.279119137 |
| 245 | Hepoxilin A3 | 319.2267 | 263.6 | 336.2301 | 12.63695 | C20H32O4 | 0.274050694 |
| 246 | Hinesol | 223.2059 | 507 | 222.1984 | 1.003558 | C15H26O | 0.262826098 |
| 247 | 4-Hydroxyphenylacetaldehyde | 136.0395 | 76 | 136.0524 | 1.148179 | C8H8O2 | 0.262589006 |
| 248 | Epiandrosterone | 271.2309 | 675 | 290.2246 | 12.27405 | C19H30O2 | 0.257406747 |
| 249 | Nicotine | 160.936 | 683.4 | 162.1157 | 9.393765 | C10H14N2 | 0.252705291 |
| 250 | Phenylethylamine | 121.0292 | 684.1 | 121.0891 | 6.870683 | C8H11N | 0.245595765 |
| 251 | 1-Methyladenosine | 282.2148 | 509.9 | 281.1124 | 13.56389 | C11H15N5O4 | 0.238831607 |
| 252 | Pentadecanoic acid | 241.2172 | 622.7 | 242.2246 | 0.431976 | C15H30O2 | 0.222854447 |
| 253 | Methyl hexadecanoic acid | 253.2534 | 494.3 | 270.2559 | 19.48246 | C17H34O2 | 0.213147701 |
| 254 | Palmitoleic acid | 254.2483 | 573.1 | 254.2246 | 4.21644 | C16H30O2 | 0.177312784 |
| 255 | 3-Geranylgeranylindole | 389.3214 | 488.6 | 389.3083 | 3.081117 | C28H39N | 0.174948178 |
| 256 | Fluconazole | 307.101 | 501 | 306.1041 | 23.69373 | C13H12F2N6O | 0.157051499 |
| 257 | Folic acid | 441.2565 | 483.3 | 441.1397 | 8.644526 | C19H19N7O6 | 0.130808841 |
| 258 | N-Acetyl-D-glucosamine | 221.1546 | 522.6 | 221.0899 | 1.819144 | C8H15NO6 | 0.125645833 |
| 259 | 9(S)-HPODE | 293.2126 | 662.4 | 312.2301 | 3.03534 | C18H32O4 | 0.095644651 |
| 260 | Spermidine | 143.9153 | 32.9 | 145.1579 | 5.514261 | C7H19N3 | 0.065557052 |
